# A combined magnetic circular dichroism and density functional theory approach for the elucidation of electronic structure and bonding in three- and four-coordinate iron(ii)–N-heterocyclic carbene complexes†
†Electronic supplementary information (ESI) available: Supplemental MCD and Mössbauer data; MO diagrams, TD-DFT, optimized geometry coordinates and X-ray crystallographic details. CCDC 1023546–1023548. For ESI and crystallographic data in CIF or other electronic format see DOI: 10.1039/c4sc02791d



**DOI:** 10.1039/c4sc02791d

**Published:** 2014-11-10

**Authors:** Kathlyn L. Fillman, Jacob A. Przyojski, Malik H. Al-Afyouni, Zachary J. Tonzetich, Michael L. Neidig

**Affiliations:** a Department of Chemistry , University of Rochester , Rochester , New York 14627 , USA . Email: neidig@chem.rochester.edu; b Department of Chemistry , University of Texas at San Antonio , San Antonio , Texas 78249 , USA

## Abstract

Iron salts and N-heterocyclic carbene (NHC) ligands is a highly effective combination in catalysis, with observed catalytic activities being highly dependent on the nature of the NHC ligand. Detailed spectroscopic and electronic structure studies have been performed on both three- and four-coordinate iron(ii)–NHC complexes using a combined magnetic circular dichroism (MCD) and density functional theory (DFT) approach that provide detailed insight into the relative ligation properties of NHCs compared to traditional phosphine and amine ligands as well as the effects of NHC backbone structural variations on iron(ii)–NHC bonding. Near-infrared MCD studies indicate that 10*Dq*(*T*_d_) for (NHC)_2_FeCl_2_ complexes is intermediate between those for comparable amine and phosphine complexes, demonstrating that such iron(ii)–NHC and iron(ii)–phosphine complexes are not simply analogues of one another. Theoretical studies including charge decomposition analysis indicate that the NHC ligands are slightly stronger donor ligands than phosphines but also result in significant weakening of the Fe–Cl bonds compared to phosphine and amine ligands. The net result is significant differences in the d orbital energies in four-coordinate (NHC)_2_FeCl_2_ complexes relative to the comparable phosphine complexes, where such electronic structure differences are likely a significant contributing factor to the differing catalytic performances observed with these ligands. Furthermore, Mössbauer, MCD and DFT studies of the effects of NHC backbone structure variations (*i.e.* saturated, unsaturated, chlorinated) on iron–NHC bonding and electronic structure in both three- and four-coordinate iron(ii)–NHC complexes indicate only small differences as a function of backbone structure, that are likely amplified at lower oxidation states of iron due to the resulting decrease in the energy separation between the occupied iron d orbitals and the unoccupied NHC π* orbitals.

## Abstract 2

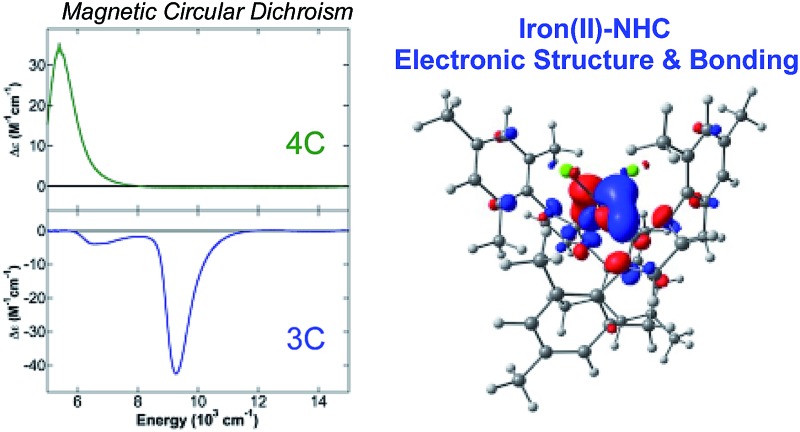
Studies of electronic structure and bonding in iron(ii)–NHC complexes using a combined magnetic circular dichroism and DFT approach.

## Introduction

During the last few decades, transition metal catalysts utilizing precious metals (*i.e.* Pd, Pt, Rh, Ir, Au, Ru) combined with NHC ligands have been successfully developed and employed for a variety of catalytic transformations including metathesis, C–H activation, hydrogenation and C–C bond formation reactions amongst many others.^1–3^ While the coordination chemistry and catalysis of late transition metal–NHC complexes have been widely explored, the development and application of iron–NHCs has only recently begun to be widely investigated despite the fact that iron–NHC complexes have been known since the early 1970's.^4–6^ Within the last decade, numerous reports of iron–NHC complexes with unique structures and novel catalytic applications have been reported.^7–22^

**Scheme 1:**
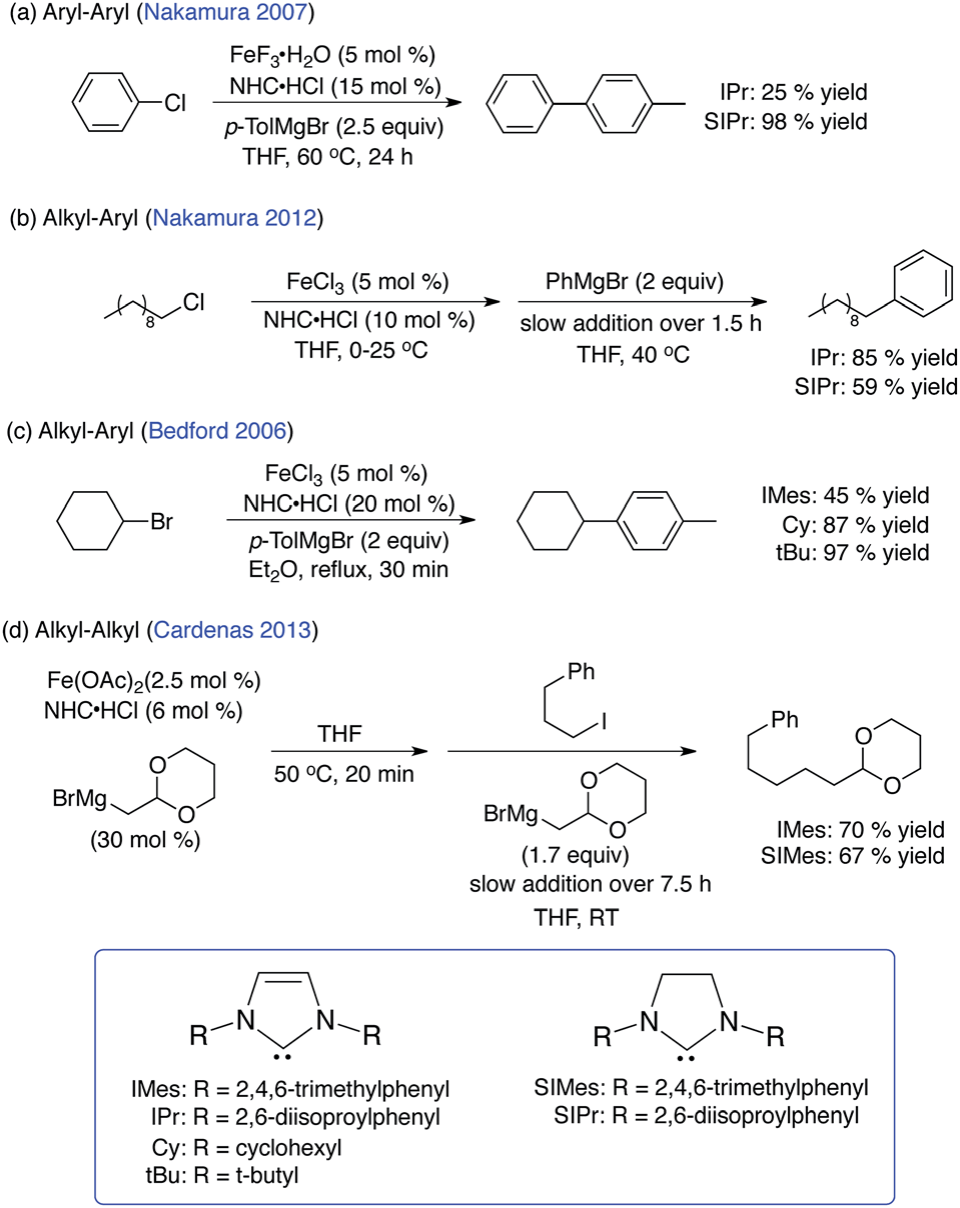
Effect of NHC ligands in iron–NHC catalyzed C–C cross-couplings.

A wide variety of applications of iron–NHC complexes to catalytic transformations have begun to emerge, including applications in hydrosilylation, carbometallation, cyclization, aziridination and allylic substitution reactions, demonstrating the significant catalytic potential of iron–NHC complexes.^7–9^ Of particular note, the combination of simple iron salts and NHCs results in the *in situ* generation of catalytically active iron–NHC species for C–C cross-coupling reactions including aryl–aryl, alkyl–aryl and alkyl–alkyl couplings (Scheme 1). NHC ligands have been particularly successful for iron cross-coupling as the aryl–aryl system is currently the best performing iron-based system for heteroaryl coupling and the alkyl–alkyl system is currently the only iron-based cross-coupling system capable of C(sp^3^)–C(sp^3^) couplings in the presence of functional groups.

Within the catalytic applications of iron–NHCs, the nature of the specific NHC ligand that provides for the most effective catalysis varies significantly from one system to another. For example, the optimal NHC for aryl–aryl cross-coupling catalysis is SIPr where the corresponding, unsaturated IPr ligand was found to be much less effective (Scheme 1).^23^ By contrast, for the cross-coupling of non-activated chloroalkanes and aryl Grignards in Kumada-type couplings, the inverse of the NHC dependence on activity is observed with IPr outperforming SIPr.^24^ However, these dramatic disparities in reactivity due to differences in NHC backbone saturation are not observed in alkyl–alkyl cross-coupling where similar product yields can be obtained using either IMes or SIMes.^25^ In the aryl–alkyl cross-coupling system of Bedford and co-workers, NHCs lacking *N*-aryl substituents (Cy, *t*Bu) outperformed IMes.^26^ Combined, these studies suggest that the NHC ring structure (*i.e.* saturated *vs.* unsaturated *vs.* substituted) and N-substitution may yield important differences in iron–NHC bonding, *in situ* iron–NHC speciation and, hence, iron–NHC reactivity.

Despite the observed dependence of catalytic performance on the NHC ligand structure, a detailed understanding of iron–NHC σ- and π-bonding and the effects of NHC ring perturbations on iron–NHC bonding is critically underdeveloped. In fact, detailed investigations of iron–NHC bonding have been limited to piano stool type iron(ii) complexes with both cyclopentadienyl (Cp) and CO ligation where a combination of IR, electrochemical and theoretical methods suggested that the NHC ligand in these complexes can serve as both a σ-donor and moderate π-acceptor.^27^ However, fundamental insight into iron–NHC bonding in more electron deficient, high-spin iron complexes is lacking. This deficiency stands in stark contrast to precious metal NHC systems where IR studies of supporting CO ligands and DFT investigations have led to the general view of NHC ligands as strong σ-donors (stronger than phosphine ligands) and weak π-acceptors, where the extent of π-bonding is dependent on the nature of the metal and supporting ligands.^28–43^ While the general views of metal–NHC bonding from the precious metal systems currently drive much of the work in iron–NHC systems, rational catalyst development with iron–NHCs necessitates a fundamental understanding of the effects of NHC variations on electronic structure and bonding in paramagnetic iron systems that may be catalytically relevant. Such studies can also provide fundamental insight into the differences in electronic structures of iron–NHC and iron–phosphine complexes lacking CO ligation that may be relevant to catalysis, including iron-catalyzed cross-coupling. Importantly, iron–NHC bonding in high-spin systems may differ significantly compared to the low-spin, Cp and CO bound species more commonly investigated.

Towards this goal, an approach combining magnetic circular dichroism (MCD) studies and density functional theory (DFT) investigations of well-defined iron(ii)–NHC complexes has been utilized to directly investigate electronic structure and bonding in high-spin iron(ii)–NHC complexes. The results provide direct insight into the ligand-field strength of NHC ligands compared to amine and phosphine ligands, the effects of NHC ring variations on bonding and the extent of donation and back donation contributions to bonding in iron(ii)–NHC complexes as a function of coordination number and geometry.

## Experimental

### General considerations

All reagents were purchased from commercial sources and used as received. Air and moisture sensitive manipulations were carried out in an MBraun inert-atmosphere (N_2_) dry box equipped with a direct liquid nitrogen inlet line or in an MBraun inert-atmosphere (Ar) dry box. All anhydrous solvents were further dried using activated alumina/4 Å molecular sieves and stored under inert-atmosphere over molecular sieves. (PPh_3_)_2_FeCl_2_, (PMe_3_)_2_FeCl_2_, (tmpn)FeCl_2_ and (teeda)FeCl_2_ were prepared following previously reported methods.^44–47^

### Synthesis of iron(ii)–NHC complexes

(IMes)_2_FeCl_2_, (IPr)Fe(CH_2_TMS)_2_, and (SIPr)Fe(CH_2_TMS)_2_ were prepared according to published procedures or slight modifications thereof.^11,13^ Related procedures for the synthesis of (^Cl^IMes)_2_FeCl_2_ and (^Cl^IPr)Fe(CH_2_TMS)_2_ appear in the ESI.†

### Mössbauer spectroscopy

All solid samples for ^57^Fe Mössbauer spectroscopy were run on non-enriched samples of the as-isolated complexes. All samples were prepared in an inert atmosphere glove box equipped with a liquid nitrogen fill port to enable sample freezing to 77 K within the glove box. Each sample was loaded into a Delrin Mössbauer sample cup for measurements and loaded under liquid nitrogen. Low temperature ^57^Fe Mössbauer measurements were performed using a See Co. MS4 Mössbauer spectrometer integrated with a Janis SVT-400 He/N_2_ cryostat for measurements at 80 K with a 0.07 T applied magnetic field. Isomer shifts were determined relative to α-Fe at 298 K. All Mössbauer spectra were fit using the program WMoss (SeeCo).

### Magnetic circular dichroism spectroscopy

All samples for MCD spectroscopy were prepared in an inert atmosphere glove box equipped with a liquid nitrogen fill port to enable sample freezing to 77 K within the glove box. MCD samples were prepared in 6 : 1 (v/v) toluene-d_8_ : benzene-d_6_ (to form low temperature optical glasses) in copper cells fitted with quartz disks and a 3 mm gasket. Low temperature MCD experiments were conducted using two Jasco spectropolarimeters. Both instruments utilize a modified sample compartment incorporating focusing optics and an Oxford Instruments SM4000-7T superconducting magnet/cryostat. This set-up permits measurements from 1.6 K to 290 K with magnetic fields up to 7 T. A calibrated Cernox sensor directly inserted in the copper sample holder is used to measure the temperature at the sample to ±0.001 K. UV-visible (UV-vis) MCD spectra were collected using a Jasco J-715 spectropolarimeter and a shielded S-20 photomultiplier tube. Near-infrared (NIR) MCD spectra were collected using a Jasco J-730 spectropolarimeter with a liquid nitrogen cooled InSb detector. The spectral range accessible with this NIR MCD setup is 2000–600 nm. All MCD spectra were baseline-corrected against zero-field scans. VTVH-MCD spectra were analyzed using previously reported fitting procedures.^48^ For VTVH-MCD fitting, both negative and positive zero-field splitting models were evaluated. The reported error bars were determined *via* evaluation of the effects of systematic variations of the fit parameters on the quality of the overall fit. *D* and |*E*/*D*| values are obtained directly from the fit parameters using the relationships *E* = (*δ*/6) + 1/3[(*δ* ^2^/2) + *δE*_s_]^1/2^ and –*D* = *E* + (*E*_s_/3) – (*δ*/6) for *S* = 2 as previously described.^48^

### Electronic structure calculations

Spin unrestricted DFT calculations were performed with the Gaussian 09 package.^49^ All geometry optimization calculations were performed with the B3LYP exchange–correlation functional^50,51^ with the TZVP^52^ basis set on all atoms and inclusion of solvation effects using the Polarized Continuum Model (PCM) with toluene as the solvent.^53^ The dispersion correction of Grimme (GD3) combined with the damping function of Becke and Johnson (BJ) was used in geometry optimizations of all four-coordinate complexes.^54^ The geometries of all complexes were fully optimized starting from X-ray crystal structures (when available) with initial optimization performed with cep-4g before optimizing at the TZVP level. All optimized geometries had frequencies found to be positive.

Further calculations of molecular orbitals (MOs) and TD-DFT used the B3LYP functional with the TZVP basis set on all atoms. MO compositions and analyses were calculated using the AOMix program.^55,56^ Atomic charges and spin densities were calculated using Mulliken population analysis (MPA). Orbitals from the Gaussian calculations were plotted with the ChemCraft program. TD-DFT was used to calculate the electronic transition energies and intensities of the 30–40 lowest-energy states. The analysis of the MO compositions in terms of fragment orbitals, Mayer bond orders, total overlap populations and the charge decomposition analysis (CDA)^57,58^ were performed using AOMix-FO.^55^ CDA and its applications have been previously described in detail by Gorelsky and co-workers.^59,60^

## Results and discussion

### Spectroscopic and electronic structure studies of (IMes)_2_FeCl_2_

**Scheme 2:**
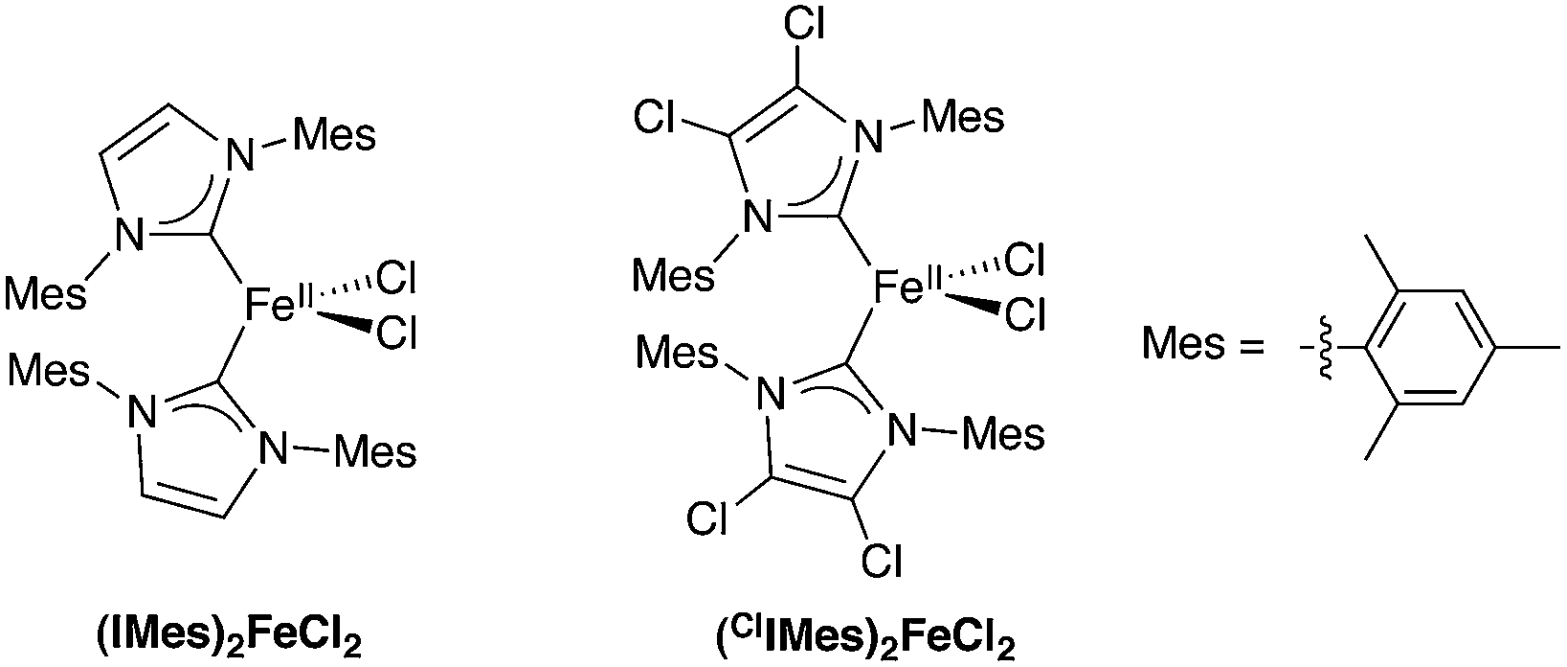
Distorted tetrahedral (NHC)_2_FeCl_2_ complexes with IMes and ^Cl^IMes ligands.

**Fig. 1:**
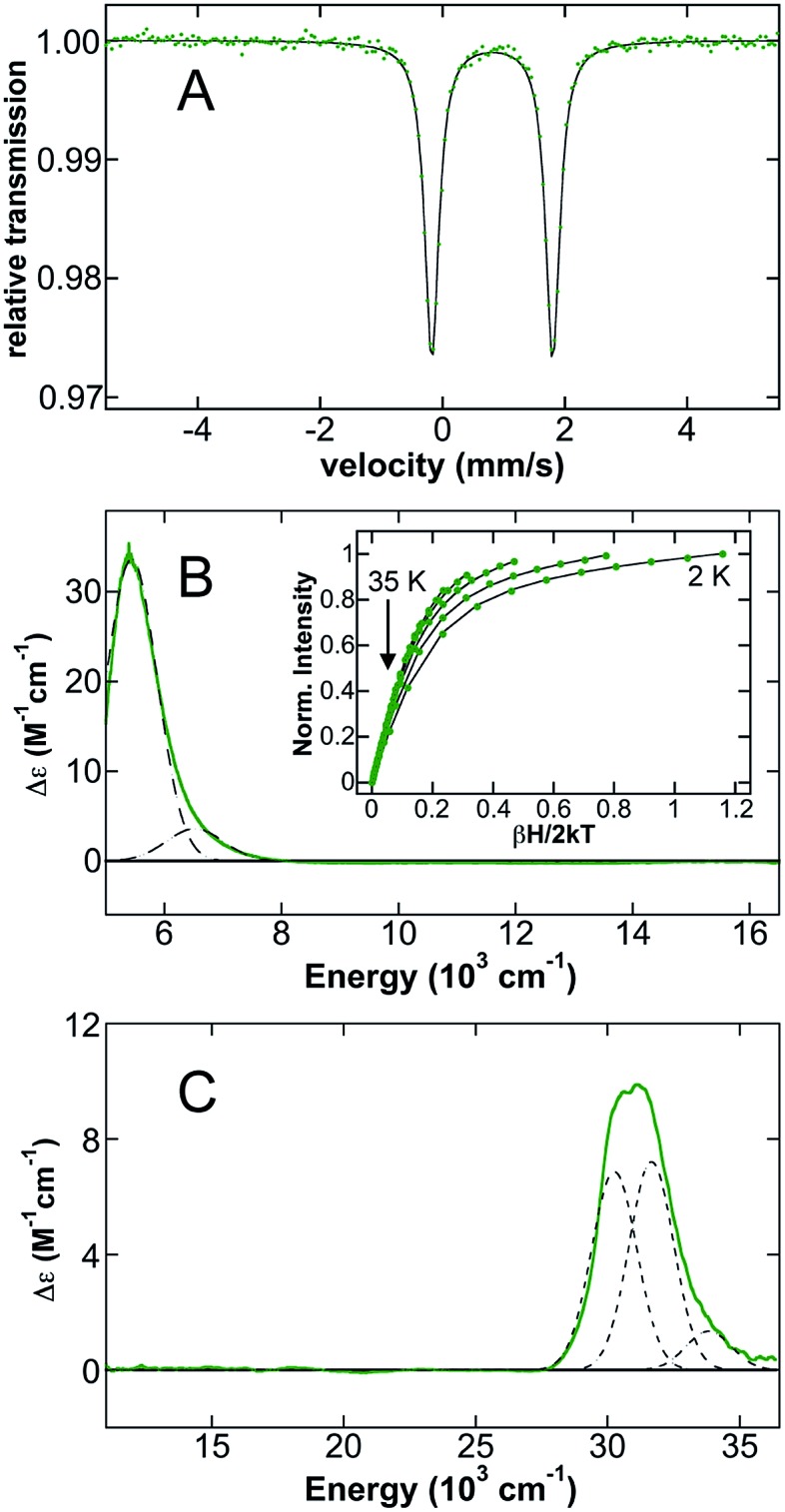
^57^Fe Mössbauer and MCD spectroscopy of (IMes)_2_FeCl_2_. (A) 80 K Mössbauer spectrum including data (dots) and fit (solid line), (B) 5 K, 7 T NIR MCD spectrum and (C) 5 K, 7 T UV-vis MCD spectrum. (B, inset) VTVH-MCD data (dots) and fit (lines) of (IMes)_2_FeCl_2_ collected at 5917 cm^–1^. MCD spectra were collected on a 3 mM solution of (IMes)_2_FeCl_2_ in 6 : 1 (toluene-d_8_ : benzene-d_6_). Peak fits are shown for the MCD spectra (dashed lines).

Initial studies focused on (IMes)_2_FeCl_2_ as a representative example of distorted tetrahedral (NHC)_2_FeX_2_ complexes which have been widely explored synthetically and as potential iron(ii) pre-catalysts for a variety of reactions (Scheme 2). The 80 K ^57^Fe Mössbauer spectrum of polycrystalline (IMes)_2_FeCl_2_ (Fig. 1A) is well-fit as a single iron species with *δ* = 0.80 mm s^–1^ and Δ*E*_Q_ = 2.12 mm s^–1^, where the observed isomer shift falls within the expected range for high-spin iron(ii), *S* = 2 distorted tetrahedral species.^61^ The 5 K, 7 T NIR MCD spectrum of (IMes)_2_FeCl_2_ contains two low-energy ligand-field (LF) transitions at 5440 cm^–1^ and 6520 cm^–1^ (10*Dq*(*T*_d_) = 5980 cm^–1^) (Fig. 1B). For a tetrahedral *S* = 2 iron(ii) complex, only the ^5^E → ^5^T_2_ transition is spin allowed. For the distorted tetrahedral environment as is present in (IMes)_2_FeCl_2_, the degeneracy of both the ground and excited states is removed, resulting in two LF transitions as observed by MCD spectroscopy. The saturation magnetization behavior for (IMes)_2_FeCl_2_ collected at 5917 cm^–1^ is well-described by a *S* = 2 negative zero-field split (–ZFS) non-Kramers doublet model with ground-state spin-Hamiltonian parameters of *δ* = 2.8 ± 0.2 cm^–1^ and *g*_∥_ = 8.5 ± 0.2 with *D* = –11 ± 1 cm^–1^ and |*E*/*D*| = 0.30 ± 0.02 (Fig. 1B, inset). The 5 K, 7 T UV-vis MCD spectrum of (IMes)_2_FeCl_2_ contains multiple charge transfer (CT) transitions in the 30 000–35 000 cm^–1^ region (Fig. 1C) which are assigned and discussed using TD-DFT calculations in the ESI.†

Spin unrestricted DFT calculations were used to further analyze the electronic structure of (IMes)_2_FeCl_2_. Geometry optimization with B3LYP/TZVP and the GD3BJ dispersion correction yielded overall structural features, bond lengths and angles in good agreement with those observed by crystallography (Table 1). The optimized (IMes)_2_FeCl_2_ complex is best described as a distorted tetrahedral complex with Fe–IMes bond lengths of 2.161 Å and 2.163 Å, Fe–Cl bond lengths of 2.334 Å, a C(IMes)–Fe–C(IMes) bond angle of 126.36° and a Cl–Fe–Cl bond angle of 107.45°. This optimized geometry correlates well with the literature structure, which has Fe–IMes bond lengths of 2.139 Å and 2.157 Å, Fe–Cl bond lengths of 2.310 Å and 2.292 Å, a C(IMes)–Fe–C(IMes) bond angle of 125.20° and a Cl–Fe–Cl bond angle of 106.66°. Both the experimental and computational studies of (IMes)_2_FeCl_2_ are indicative of a high-spin iron(ii) complex (*S* = 2). The molecular orbitals and their corresponding energies as well as electronic transition energies were calculated from the optimized structure.

**Table 1:** Comparison of experimental and calculated structural parameters of four-coordinate (4C) L_2_FeCl_2_ complexes

Complex	Fe–L_1_ (Å)		Fe–L_2_ (Å)		Fe–Cl_1_ (Å)		Fe–Cl_2_ (Å)		L_1_–Fe–L_2_ (°)	
	Exp.	Calc.	Exp.	Calc.	Exp.	Calc.	Exp.	Calc.	Exp.	Calc.
(IMes)_2_FeCl_2_	2.139	2.161	2.157	2.163	2.310	2.334	2.292	2.334	125.20	126.36
(tmpn)FeCl_2_	2.140	2.187	2.140	2.187	2.271	2.296	2.239	2.275	99.35	97.04
(teeda)FeCl_2_	2.192	2.229	2.150	2.204	2.230	2.268	2.240	2.284	83.75	83.60
(PMe_3_)_2_FeCl_2_	2.430	2.449	2.427	2.445	2.240	2.280	2.235	2.278	102.78	104.43
(PPh_3_)_2_FeCl_2_	2.476	2.482	2.476	2.482	2.219	2.271	2.219	2.271	111.25	110.07

**Fig. 2:**
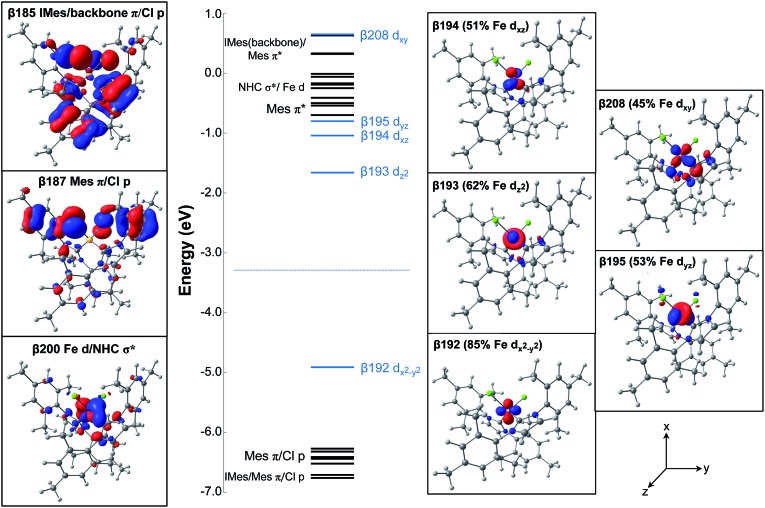
Calculated molecular orbital energy diagram for (IMes)_2_FeCl_2_.

The electronic ground state of (IMes)_2_FeCl_2_ is described by the frontier molecular orbitals (FMOs) with focus on the unoccupied MOs, in conjunction with their occupied counterparts, to showcase the major contributions to bonding. The MOs and the corresponding energy diagram are shown in Fig. 2. In the β manifold, the highest occupied molecular orbital (HOMO) along with the unoccupied orbitals β-193, β-194, β-195, and β-208 are comprised mostly of Fe d contributions, slightly mixed with NHC and Cl orbital contributions. The Fe 3d orbitals listed in order of increasing energy are d_*x*^2^–*y*^2^_ (β-192), d_*z*^2^_ (β-193), d_*xz*_ (β-194), d_*yz*_ (β-195), d_*xy*_ (β-208). In addition, there are FMOs that represent an occupied MO of both IMes (with backbone contributions) and Cl character (β-185), an occupied MO of Mes (NHC side chains) and Cl character (β-187), and an unoccupied MO with some d character as well as a σ bond with IMes (β-200).

**Fig. 3:**
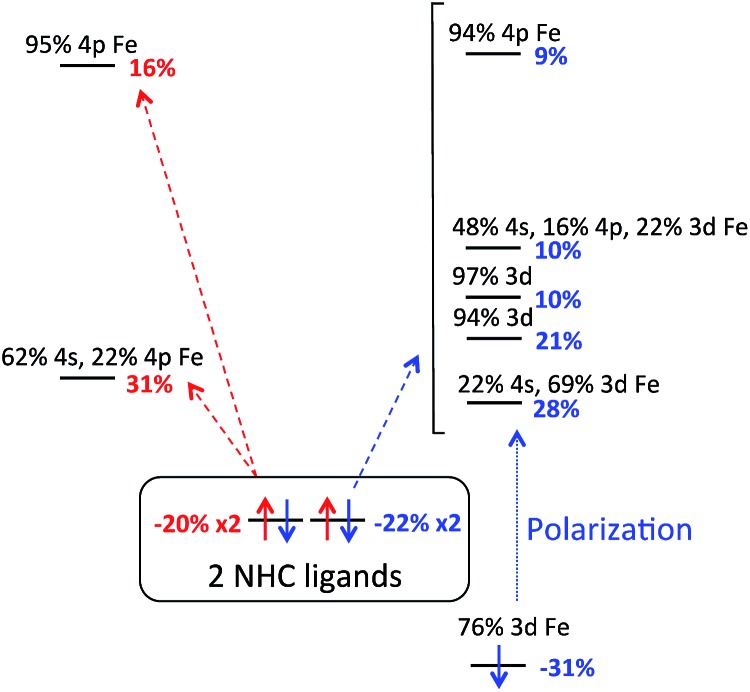
FO analysis of charge donation in (IMes)_2_FeCl_2_. Occupancy changes (α (red) and β (blue)) and orbital compositions are indicated for each FO. Arrows indicate donation to Fe–Cl_2_ FOs.

Charge decomposition (CDA), fragment molecular orbital (FO) and Mayer bond order (MBO) analyses were completed for the (IMes)_2_FeCl_2_ complex. The MOs of a complex can be described as linear combinations of the occupied and unoccupied MOs of defined molecular fragments, termed fragment molecular orbitals or FOs. In CDA, donation and back donation between different molecular fragments can be evaluated based on the overlap and coefficients of the MO–LCFO matrix (where LCFO = linear combination of fragment molecular orbitals). In the case of (IMes)_2_FeCl_2_, CDA was completed to quantify the total charge donation and back donation between the Fe–Cl_2_ fragment and two IMes ligand fragments (*i.e.* 3 total fragments). When the Fe–Cl_2_ fragment and IMes ligand fragments are combined to form the complex, there is a mixing of the occupied fragment orbital of the donor with the unoccupied fragment orbital of the acceptor which allows for the transfer of electron density (charge donation) from the donor to the acceptor. From the CDA, it can be seen that there is a total charge donation (IMes_2_ → Fe–Cl_2_) of 0.954 electrons and total back donation (Fe–Cl_2_ → IMes_2_) of 0.391 electrons. This results in a net charge donation to Fe–Cl_2_ of 0.563 electrons. The FO analysis provides information about changes in occupancies of the fragment molecular orbitals of the donor and acceptor upon the complex formation so that the orbitals involved in the donation and back donation can be readily identified (*i.e.* the specific orbitals that change their occupancies). In the α manifold, the charge donation from the IMes ligand fragments to the Fe–Cl_2_ fragment occurs from the highest occupied fragment orbital (HOFO) of the IMes fragments, an IMes σ orbital, to two unoccupied fragment orbitals on the Fe–Cl_2_ fragment of iron 4s and 4p character (20% occupancy change per NHC ligand, see Fig. 3). A slightly larger occupancy change is observed for charge donation in the β manifold (22% per NHC ligand), where donation occurs again from the HOFO (IMes σ orbital) of the IMes fragments to several unoccupied FOs of the Fe–Cl_2_ fragment that have significant iron d character. In the β manifold, there is also a significant electronic polarization, particularly in the Fe–Cl_2_ fragment, which allows for a redistribution of charge from an occupied Fe–Cl_2_ fragment orbital with mostly d character to unoccupied fragment orbitals of the Fe–Cl_2_ fragment (see Fig. 3). Furthermore, the FO analysis indicates the presence of back donation in both the α and β manifolds from occupied Fe–Cl_2_ fragment orbitals to unoccupied IMes fragment orbitals (*i.e.* ∼9.6% and ∼10.1% occupancy changes for each IMes fragment in both the α and β manifolds, respectively). However, in contrast to the donation in this system, the occupancy changes corresponding to back donation are spread over many occupied Fe–Cl_2_ and unoccupied IMes FOs. For this distorted tetrahedral complex, the Mayer bond order (MBO) between Fe and each IMes is 0.760 and 0.767. In both cases, the β contribution to the bond order is greater than that of the α, 0.390 and 0.393 for β and 0.370 and 0.374 for α. When compared to the occupied α-spin MOs, the occupied β-spin MOs contribute more to the overall bond order due to a small increase in σ donation from the NHCs to the unoccupied β-spin orbitals on Fe.

### Evaluation of the relative ligand field strengths of NHC ligands in L_2_FeCl_2_ distorted tetrahedral iron(ii) complexes

A series of distorted tetrahedral iron(ii) complexes containing two chloride ligands and two additional ligands (*e.g.* diamine, two monodentate phosphines or NHCs) were investigated by NIR MCD spectroscopy in order to evaluate the relative LF strength of NHC ligands in distorted tetrahedral iron(ii) complexes. Such an evaluation is important as it has been previously suggested that NHCs may be stronger field ligands than phosphines and, hence, may be able to serve as alternatives for phosphines in the development of iron-based catalysts.^7,8,26,27,62,63^

**Fig. 4:**
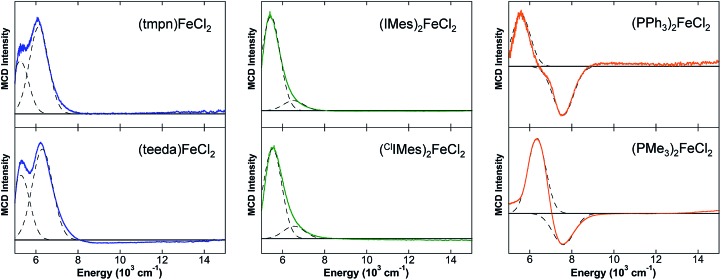
5 K, 7 T NIR MCD spectra of distorted tetrahedral L_2_FeCl_2_ complexes. Peak fits are shown for each spectrum (dashed lines). The NHC and diamine complex spectra were collected on 3 mM solutions in 6 : 1 toluene-d_8_ : benzene-d_6_. The spectra of the phosphine complexes were collected on solid state mulls of crystalline samples.

The 5 K, 7 T NIR MCD spectra of the L_2_FeCl_2_ complexes investigated are given in Fig. 4 and the d–d transition energies and 10*Dq*(*T*_d_) values are summarized in Table 2. The diamine–iron(ii)–dichloride complex (tmpn)FeCl_2_ (tmpn = *N*,*N*,*N*′,*N*′-tetramethylpropane-1,3-diamine) contains very low energy LF transitions at 5260 and 6140 cm^–1^ (10*Dq*(*T*_d_) = 5700 cm^–1^). Similarly, (teeda)FeCl_2_ (teeda = *N*,*N*,*N*′,*N*′-tetraethylethylenediamine) gives LF transitions at 5290 and 6310 cm^–1^ (10*Dq*(*T*_d_) = 5800 cm^–1^). Both NHC ligated tetrahedra gave slightly higher energy LF bands, where (^Cl^IMes)_2_FeCl_2_ exhibited LF transitions at 5520 cm^–1^ and 6540 cm^–1^ (10*Dq*(*T*_d_) = 6030 cm^–1^), similar to those observed for (IMes)_2_FeCl_2_ (10*Dq*(*T*_d_) = 5980 cm^–1^). The phosphine–iron(ii)–dichloride complexes yielded the highest energy ligand-field transitions, with (PPh_3_)_2_FeCl_2_ exhibiting LF bands at 5590 cm^–1^ and 7590 cm^–1^ (10*Dq*(*T*_d_) = 6590 cm^–1^) and (PMe_3_)_2_FeCl_2_ at 6340 cm^–1^ and 7600 cm^–1^ (10*Dq*(*T*_d_) = 6970 cm^–1^). In contrast to the NHC and diamine complexes, both phosphine species exhibit a pseudo-*A* term in their LF MCD transitions, where such pseudo-*A* terms in MCD (*i.e.* a pair of temperature-dependent C-terms with opposite sign) arise from spin orbit coupling (SOC) between two excited states that are close in energy to which two orthogonal transitions occur from a single ground state.^64–66^ While the physical origin of the pseudo-*A* term is currently elusive in the phosphine complexes as it requires a detailed understanding of the SOC mechanism, it is noteworthy that previous MCD studies of distorted tetrahedral bisphosphine complexes have also exhibited pseudo-*A* term LF transitions^67^ and the generality and origin of this behavior for tetrahedral phosphine complexes will be a focus of future study.

**Table 2:** LF transitions of 10*Dq*(*T*_d_) values for L_2_FeCl_2_ complexes

Complex	LF transitions	10*Dq*(*T*_d_)
(tmpn)FeCl_2_	5260, 6140 cm^–1^	5700 cm^–1^
(teeda)FeCl_2_	5290, 6310 cm^–1^	5800 cm^–1^
(IMes)_2_FeCl_2_	5440, 6520 cm^–1^	5980 cm^–1^
(^Cl^IMes)_2_FeCl_2_	5520, 6540 cm^–1^	6030 cm^–1^
(PPh_3_)_2_FeCl_2_	5590, 7590 cm^–1^	6590 cm^–1^
(PMe_3_)FeCl_2_	6340, 7600 cm^–1^	6970 cm^–1^

To obtain further insight into iron(ii)–NHC bonding compared to phosphine and amine ligands, CDA and MBO analyses were completed for the L_2_FeCl_2_ complexes. The MBO between Fe and the NHCs in (IMes)_2_FeCl_2_ is the highest of the series (see Table 3). Notably, the MBOs between Fe and PPh_3_ or PMe_3_ are slightly smaller. Lower still are the MBOs for the Fe–diamine bonds in (tmpn)FeCl_2_ and (teeda)FeCl_2_, 0.362 and 0.362 for (tmpn)FeCl_2_ and 0.352 and 0.336 for (teeda)FeCl_2_. However, the MBO between Fe and Cl is much higher in both the phosphine and diamine complexes when compared to the NHC complex. This is indicative of a strong *trans*-type influence of the NHC ligand that causes a pronounced weakening of the Fe–Cl bond. This influence can be seen experimentally in the (IMes)_2_FeCl_2_ crystal structure where the Fe–Cl bonds are elongated (avg Fe–Cl = 2.30 Å) in comparison to the analogous bisphosphine (avg Fe–Cl = 2.23 Å) and diamine (avg Fe–Cl = 2.26 Å) complexes (see Table 1). The calculated total overlap population (TOP) between the Fe and Cl moieties in (IMes)_2_FeCl_2_ (avg Fe–Cl = 0.39) is significantly lower than those calculated for the bisphosphine and diamine complexes (avg Fe–Cl = 0.49 and 0.50, respectively). The amount of electron density between the Fe and Cl atoms in each complex displays an inverse relationship with the amount of electron density donated by each L-type ligand (Table 3). This correlation suggests that the strongest donor to the distorted tetrahedral Fe(ii) center will cause the most pronounced weakening of the Fe–Cl bond. This rationalization using TOP values is consistent with the perturbation approach of Burdett and Albright,^68^ which shows that systems of lowered symmetry can, through orbital mixing, exhibit metal–ligand bond weakening effects similar to the *trans* influence typically seen in square-planar and octahedral complexes. The results of the CDA show that the complex with the highest net charge donation to the Fe–Cl_2_ fragment from the L-type ligand fragments is (IMes)_2_FeCl_2_ with a net charge donation of 0.563 electrons, followed by the two phosphine complexes, (PMe_3_)_2_FeCl_2_ and (PPh_3_)_2_FeCl_2_, with total net charge donations of 0.493 and 0.472 electrons, respectively. The diamine complexes, (tmpn)FeCl_2_ and (teeda)FeCl_2_, have comparatively lower total net charge donations of 0.275 and 0.250 electrons, respectively. In addition, there is significant back donation in (IMes)_2_FeCl_2_, 0.391 electrons, as well as (PMe_3_)_2_FeCl_2_ and (PPh_3_)_2_FeCl_2_, 0.222 and 0.294 electrons, respectively.

**Table 3:** Mayer bond order and charge decomposition analyses for 4C NHC, phosphine and diamine L_2_FeCl_2_ complexes

Complex	Mayer bond order		Charge decomposition analysis (α + β)		
	Fe–L	Fe–Cl	Donation: (L_2_ → Fe–Cl_2_)	Back donation: (Fe–Cl_2_ → L_2_)	Net charge donation to Fe–Cl_2_
(IMes)_2_FeCl_2_	0.760	0.659	0.954 e^–^	0.391 e^–^	0.563 e^–^
	0.767	0.660			
					
(tmpn)FeCl_2_	0.362	0.752	0.427 e^–^	0.152 e^–^	0.275 e^–^
	0.362	0.792			
					
(teeda)FeCl_2_	0.352	0.763	0.424 e^–^	0.174 e^–^	0.250 e^–^
	0.336	0.797			
					
(PMe_3_)_2_FeCl_2_	0.644	0.776	0.715 e^–^	0.222 e^–^	0.493 e^–^
	0.641	0.774			
					
(PPh_3_)_2_FeCl_2_	0.663	0.799	0.766 e^–^	0.294 e^–^	0.472 e^–^
	0.663	0.799			

### Spectroscopic and electronic structure studies of three-coordinate (NHC)Fe(CH_2_TMS)_2_ complexes

**Scheme 3:**
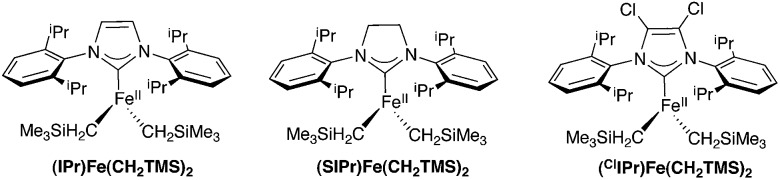
(NHC)Fe(CH_2_TMS)_2_ complexes.

**Fig. 5:**
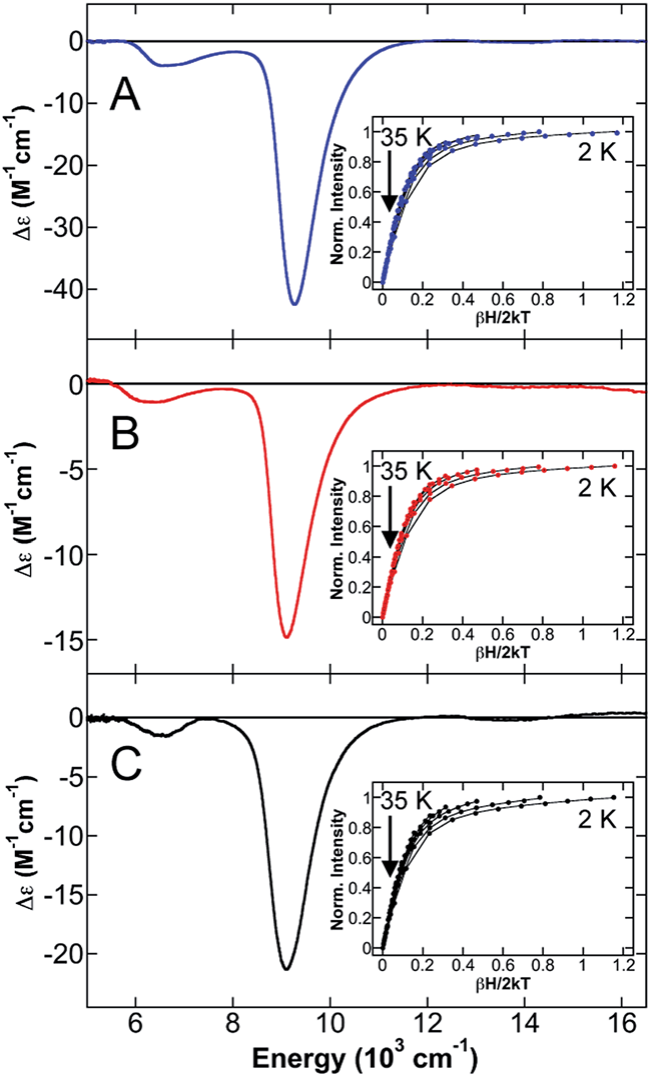
NIR MCD spectroscopy of (NHC)Fe(CH_2_TMS)_2_ complexes. 5 K, 7 T NIR MCD spectra of (A) (IPr)Fe(CH_2_TMS)_2_, (B) (SIPr)Fe(CH_2_TMS)_2_ and (C) (^Cl^IPr)Fe(CH_2_TMS)_2_. VTVH-MCD data (dots) and fit (lines) of (A, inset) (IPr)Fe(CH_2_TMS)_2_ collected at 9852 cm^–1^, (B, inset) (SIPr)Fe(CH_2_TMS)_2_ collected at 9570 cm^–1^ and (C, inset) (^Cl^IPr)Fe(CH_2_TMS)_2_ collected at 9434 cm^–1^. All MCD spectra were collected on 3 mM solutions in 6 : 1 toluene-d_8_ : benzene-d_6_.

To further evaluate the effects of NHC backbone substitutions on iron(ii)–NHC complex electronic structure and bonding as a function of coordination number and geometry, a series of three-coordinate (3C) (NHC)Fe(CH_2_TMS)_2_ complexes were studied which vary only in the nature of the NHC backbone (saturated (SIPr), unsaturated (IPr) and unsaturated/chlorinated (^Cl^IPr)) (Scheme 3). The 80 K ^57^Fe Mössbauer spectrum of solid (IPr)Fe(CH_2_TMS)_2_ (ESI, Fig. S3†) is well-fit as a single iron species with *δ* = 0.34 mm s^–1^ and Δ*E*_Q_ = 1.04 mm s^–1^. The 80 K Mössbauer spectra of solid (SIPr)Fe(CH_2_TMS)_2_ and (^Cl^IPr)Fe(CH_2_TMS)_2_ (see ESI, Fig. S3†) are very similar to (IPr)Fe(CH_2_TMS)_2_ (*δ* = 0.35 mm s^–1^, Δ*E*_Q_ = 1.12 mm s^–1^ and *δ* = 0.33 mm s^–1^, Δ*E*_Q_ = 1.08 mm s^–1^, respectively). The observed isomer shifts for these 3C species are somewhat lower than previously observed for 3C high-spin iron(ii) complexes (*δ* = 0.51 mm s^–1^ for (*a*IPr)Fe{N(SiMe_3_)_2_}_2_,^19^ 0.59 mm s^–1^ for [Li(15-crown-5)][Fe{N(SiMe_3_)_2_}_3_],^69^ 0.48 mm s^–1^ and 0.74 mm s^–1^ for CH_3_^–^ and Cl^–^ ligated iron(ii)-β-diketiminates).^70^ The NIR MCD spectrum of (IPr)Fe(CH_2_TMS)_2_ contains two LF transitions at 6660 cm^–1^ and 9260 cm^–1^ (Fig. 5A). The saturation magnetization behavior for (IPr)Fe(CH_2_TMS)_2_ collected at 9852 cm^–1^ is well-described by a *S* = 2 –ZFS non-Kramers doublet model with ground-state spin-Hamiltonian parameters of *δ* = 2.1 ± 0.2 cm^–1^ and *g*_ll_ = 9.9 ± 0.2 with *D* = –20 ± 2 cm^–1^ and |*E*/*D*| = 0.20 ± 0.02 (Fig. 5A, inset). The observed *D* value is similar to those previously determined for (IPr)Fe{N(SiMe_3_)_2_}_2_ (*D* = –18.2 cm^–1^) and (IMes)Fe{N(SiMe_3_)_2_}_2_ (*D* = –23.3 cm^–1^) by Layfield and co-workers.^71^ The 5 K, 7 T NIR MCD spectra of (SIPr)Fe(CH_2_TMS)_2_ (Fig. 5B) and (^Cl^IPr)Fe(CH_2_TMS)_2_ (Fig. 5C) yielded similar LF transitions and ZFS parameters to (IPr)Fe(CH_2_TMS)_2_ (Table 4). Thus, NIR MCD spectroscopy supports the presence of similar electronic structures in the 3C (NHC)Fe(CH_2_TMS)_2_ complexes. Lastly, UV-vis MCD spectra of the three complexes indicate small differences in the CT transitions (see ESI†).

**Table 4:** Mössbauer, LF and ZFS parameters for 3C (NHC)Fe(CH_2_TMS)_2_ complexes

Complex	Mössbauer		LF bands (cm^–1^)	ZFS parameters		
	*δ* (mm s^–1^)	Δ*E*_Q_ (mm s^–1^)		*D* (cm^–1^)	|*E*|(cm^–1^)	|*E*/*D*|
(IPr)Fe(CH_2_TMS)_2_	0.34	1.04	6660, 9260	–20 ± 2	3.8 ± 0.5	0.19 ± 0.02
(SIPr)Fe(CH_2_TMS)_2_	0.35	1.12	6350, 9110	–20 ± 2	4 ± 0.5	0.20 ± 0.02
(^CI^IPr)Fe(CH_2_TMS)_2_	0.33	1.08	6520, 9100	–18 ± 2	3.4 ± 0.5	0.19 ± 0.02

Spin unrestricted DFT calculations were used to further analyze the electronic structures of (IPr)Fe(CH_2_TMS)_2_, (SIPr)Fe(CH_2_TMS)_2_, and (^Cl^IPr)Fe(CH_2_TMS)_2_. Geometry optimizations with B3LYP/TZVP yielded overall structural features, bond lengths and angles in good agreement with those observed by crystallography (Table 5; note that no crystal structure is available for (^Cl^IPr)Fe(CH_2_TMS)_2_). Both experimental and computational studies of the series of 3C NHC complexes are indicative of high-spin iron(ii) complexes (*S* = 2). Details of the MO analyses are given in the ESI.† Importantly, CDA and MBO analyses were completed for the series of 3C Fe(ii) NHC complexes (Table 6). From the CDA, it can be seen that the total donation from the NHC fragment to the Fe–(CH_2_TMS)_2_ fragment is the highest for (IPr)Fe(CH_2_TMS)_2_, suggesting that this complex is the strongest σ-donor of the series, followed by (SIPr)Fe(CH_2_TMS)_2_, and (^Cl^IPr)Fe(CH_2_TMS)_2_ (see Table 6). While it can also be seen that (SIPr)Fe(CH_2_TMS)_2_ has the highest back donation of the series, the overall back donation to the NHC fragment (average = 0.076 electrons) for all three complexes is small when compared to the total donation to the Fe–(CH_2_TMS)_2_ fragment (average = 0.444 electrons). For (IPr)Fe(CH_2_TMS)_2_, the MBO between Fe and IPr is 0.667, with α and β contributions of 0.341 and 0.326, respectively. Similarly, the MBO between Fe and the NHC for (SIPr)Fe(CH_2_TMS)_2_ and (^Cl^IPr)Fe(CH_2_TMS)_2_ are 0.664 and 0.625, respectively. The small disparities in the σ donation abilities of the NHC ligands in the 3C complexes derive from slightly different MBOs of the Fe–C(NHC) bonds and the C(NHC)–N(NHC) bonds. In (SIPr)Fe(CH_2_TMS)_2_, the C(NHC)–N(NHC) bonds are relatively strong (average MBO = 1.168) which weakens the Fe–C(NHC) bond, thereby making it a poorer σ donor. The C(NHC)–N(NHC) bond in (IPr)Fe(CH_2_TMS)_2_ is weaker than that of the SIPr complex (average MBO = 1.130), consequently causing a stronger Fe–C(NHC) bond and making the IPr complex an overall better σ donor. Overall, while slight differences in iron–NHC bonding are present as a function of the NHC backbone structure, both experimental and theoretical studies indicate that these differences are relatively small in this series of three-coordinate iron(ii) complexes. In contrast to previous proposals that backbone substituents can greatly affect bonding interactions in Fe–NHC complexes,^7^ this result is consistent with our studies of 4C complexes where the electronic structure effects of IMes *vs.* ^Cl^IMes are minimal for *S* = 2 iron(ii).

**Table 5:** Comparison of experimental and calculated structural parameters for 3C (NHC)Fe(CH_2_TMS)_2_ complexes

Complex	Fe–NHC (Å)		Fe–(CH_2_TMS)_1_ (Å)		Fe–(CH_2_TMS)_2_ (Å)		(CH_2_TMS)_1_–Fe–(CH_2_TMS)_2_ (°)		NHC–Fe–(CH_2_TMS)_1_ (°)	
	Exp.	Calc.	Exp.	Calc.	Exp.	Calc.	Exp.	Calc.	Exp.	Calc.
(IPr)Fe(CH_2_TMS)_2_	2.164	2.182	2.060	2.096	2.062	2.096	123.09	126.06	118.46	116.97
(SIPr)Fe(CH_2_TMS)_2_	2.177	2.201	2.056	2.095	2.056	2.095	122.89	126.04	118.56	116.98
(^CI^IPr)Fe(CH_2_TMS)_2_	—	2.234	—	2.080	—	2.080	—	117.96	—	121.02

**Table 6:** Mayer bond order and charge decomposition analyses for 3C (NHC)Fe(CH_2_TMS)_2_ complexes

Complex	Mayer bond order analysis			Charge decomposition analysis (α + β)		
	Fe–NHC	α contr.	β contr.	Donation: (NHC → Fe–(CH_2_TMS)_2_)	Back donation: (Fe–(CH_2_TMS)_2_ → NHC)	Net charge donation to Fe–(CH_2_TMS)_2_
(IPr)Fe(CH_2_TMS)_2_	0.667	0.341	0.326	0.470 e^–^	0.068 e^–^	0.402 e^–^
(SIPr)Fe(CH_2_TMS)_2_	0.664	0.336	0.327	0.439 e^–^	0.082 e^–^	0.357 e^–^
(^CI^IPr)Fe(CH_2_TMS)_2_	0.625	0.330	0.295	0.422 e^–^	0.077 e^–^	0.345 e^–^

## Conclusions

While significant progress has been made in the understanding of metal–NHC bonding and electronic structure in precious metal systems, especially electron rich systems with CO ligation, the elucidation of electronic structure and bonding in high-spin iron–NHC systems remains limited. In the present study, the first application of an approach combining magnetic circular dichroism studies to evaluate LF transitions, 10*Dq*(*T*_d_) and metal-centered charge-transfer transitions with detailed DFT studies including Mayer bond order and charge decomposition analyses is used to evaluate electronic structure and bonding in electron poor iron–NHCs. In contrast to IR-based methods reliant upon CO ligation, this approach is a direct probe of electronic structure in iron–NHC complexes and broadly applicable to any *S* > 0 iron–NHC species and, hence, is not limited to the low-spin iron species generally present with CO ligation. In terms of catalysis, the ability to probe electronic structure and bonding in more electron deficient iron species is essential as it has been proposed that such iron(i) and iron(ii) species may be active in cross-coupling.

Near-infrared MCD studies of distorted tetrahedral (IMes)_2_FeCl_2_ compared to a series of L_2_FeCl_2_ distorted tetrahedral (L = phosphine or amine) permit *the first direct elucidation of 10Dq(T*_*d*_ *) and hence, ligand field strength, of NHCs relative to phosphine and diamine ligands.* From these studies, 10*Dq*(*T*_d_) for (IMes)_2_FeCl_2_ is found to be intermediate in magnitude relative to the corresponding phosphine (largest 10*Dq*(*T*_d_)) and diamine (smallest 10*Dq*(*T*_d_)) complexes. While the observed 10*Dq*(*T*_d_) values initially appear to contradict existing views of NHCs as stronger field ligands than phosphines,^35,72^ Mayer bond order and charge decomposition analyses indicate that the NHC is a stronger donor ligand than phosphines. The origin of the reduced 10*Dq*(*T*_d_) value with NHC ligation is found to reflect the significant weakening of the Fe–Cl bonds (leading to reduced Fe–Cl bond orders and reduced charge donation from Cl to Fe) in the NHC complexes relative to the comparable amine and phosphine complexes. While strong *trans*-type influences in metal–NHCs have been previously proposed,^72^ this study provides the first direct quantitative evaluation of this in terms of the resulting energies of both the occupied and unoccupied d orbitals. These effects are significant in L_2_FeCl_2_ complexes as indicated by the large differences in 10*Dq*(*T*_d_), *clearly demonstrating that NHCs are not simple analogues of phosphine ligands for iron.* Importantly, the differences in the electronic structures of iron–NHC *vs.* iron–phosphine complexes are likely a significant contributing factor to the differing catalytic performances observed with these ligands. For example, the variations in the d orbital energies (both occupied and unoccupied) could directly affect the reaction barriers for homolytic R–X cleavage or oxidative addition proposed in iron-catalyzed cross-coupling.^73–75^ Furthermore, the significant effect of iron–NHC coordination on the ligation strength of other ligands to iron, such as nucleophile-derived ligands in cross-coupling, could provide a pathway to modulate the reactivity of coordinated ligands that ultimately form new C–C bonds upon reaction with electrophiles. For example, it is anticipated that the *trans*-type influence due to the NHC ligation will result in a *trans* effect where the rate of Fe–Cl substitution/transmetalation will be increased. Similarly, for transmetalated iron species of the form (IMes)_2_FeRX or (IMes)_2_FeR_2_, the rate of Fe–R bond dissociation required for cross-coupled product generation should also be increased due to this effect.

Analogous studies of electronic structure and bonding as a function of NHC backbone structure (*e.g.* saturated, unsaturated, chlorinated) in both three- and four-coordinate iron(ii)–NHC complexes were also performed due to the significant differences reported in catalytic systems as a function of NHC backbone structure. In these high-spin iron(ii) systems (*S* = 2), minimal effects were observed as a function of NHC backbone structure. However, the small differences in back donation observed are likely amplified at lower oxidation states of iron due to the resulting decrease in the energy separation between the occupied iron d orbitals and the unoccupied NHC π* orbitals as the iron oxidation state is reduced below iron(ii). While the iron(i)–NHC complex, (IMes)_2_FeCl, has been recently reported in the literature,^76^ iron(i)–NHC complexes with varied NHC backbone structures are not yet known. However, preliminary DFT and spectroscopic studies of (IMes)_2_FeCl are consistent with a significant increase in Fe–Cl → IMes_2_ backdonation upon reduction to iron(i). Once iron(i) complexes with varied NHC ligands are synthetically accessible, electronic structure studies can provide further insight into the effects of NHC structure on iron–NHC bonding as a function of iron oxidation state.

The continued application of the combined MCD and DFT approach employed herein to additional iron–NHC systems, including N-substituent variations in iron(ii)–NHCs and iron(i)–NHCs as a function of coordination number, geometry and supporting ligands, should continue to expand our fundamental understanding of iron–NHC electronic structure and bonding. Ultimately, such studies will continue to provide critical insight into the molecular-level origins of variations in catalytic performance as a function of NHC structure with catalytically relevant supporting ligands.
