# Supplementary material for: A combined magnetic circular dichroism and density functional theory approach for the elucidation of electronic structure and bonding in three- and four-coordinate iron(ii)–N-heterocyclic carbene complexes
Source: Chem Sci. 2014 Nov 10;6(2):1178–88. doi: 10.1039/c4sc02791d (PMC4302958; doi:10.1039/c4sc02791d)
Supplement: Supplementary file 1 [file SC-006-C4SC02791D-s001.pdf]

Electronic Supplementary Material (ESI) for Chemical Science.
This journal is © The Royal Society of Chemistry 2014

Supporting Information for

A combined magnetic circular dichroism and density functional theory approach for the elucidation of electronic structure and bonding in three- and four-coordinate iron(II)-*N*-heterocyclic carbene complexes

Kathlyn L. Fillman,[a] Jacob A. Przyojski,[b] Malik H. Al-Afyouni,[a] Zachary J. Tonzetich[b] and Michael L. Neidig*[a]

[a] Department of Chemistry, University of Rochester, Rochester, New York 14627, USA

[b] Department of Chemistry, University of Texas at San Antonio, San Antonio, Texas 78249, USA

Table of Contents

1. Experimental S2
    1.1 Synthetic Procedures.. S2
    1.2 NMR Spectra.. S3
2. Supplementary Data S5
    2.1 Mössbauer Spectra S5
    2.2 UV-Visible MCD Spectra S6
3. DFT Studies S7
    3.1 MO Energy Level Diagrams S7
    3.2 TD-DFT S10
    3.3 Optimized Geometry Coordinates S12
4. X-ray Crystallography S25
    4.1 Notes on Structure Refinement S25
    4.2 Thermal Ellipsoid Drawings S26
    4.3 Crystallographic Data and Refinement Parameters S29

1. Experimental

1.1 Synthetic Procedures

General Comments. All manipulations were performed under an atmosphere of nitrogen gas using standard Schlenk technique or in a Vacuum Atmospheres glovebox. Tetrahydrofuran, diethyl ether, pentane, and toluene were purified by sparging with argon and passage through two columns packed with 4 Å molecular sieves. Benzene-d_6 was dried over sodium ketyl and vacuum-distilled prior to use. NMR spectra were recorded in benzene-d_6 on a Varian spectrometer operating at 500 MHz (^{1}H). Chemical shift values were referenced to the residual ^{1}H (7.16 ppm) resonance of solvent. IPr, ^{Cl}IMes, ^{Cl}IPr, (IMes)$_2$FeCl$_2$, [(IPr)FeCl(μ-Cl)]$_2$, [(SIPr)FeCl(μ-Cl)]$_2$, and [(^{Cl}IPr)FeCl(μ-Cl)]$_2$ were prepared by published procedures.[1 2 3] Modified procedures for (IPr)Fe(CH$_2$TMS)$_2$ and (SIPr)Fe(CH$_2$TMS)$_2$, reported previously by Danopoulos and Braunstein,[3] are included below.

(^{Cl}IMes)$_2$FeCl$_2$. A flask was charged with 270 mg (724 μmol) of ^{Cl}IMes and 15 mL of THF. To the stirring solution was added 84.3 mg (362 μmol) of FeCl$_2$(THF)$_{1.5}$. The golden colored solution was allowed to stir at ambient temperature for 1 h. All volatiles were removed in vacuo, and the resulting residue was extracted into 10 mL of warm toluene. The toluene solution was filtered and chilled to −30° C for 20 h during which time the desired compound precipitated as 272 mg (77%) of off-white crystals. Crystals suitable for X-ray diffraction were grown by vapor diffusion of pentane into a saturated benzene solution. ^{1}H NMR: δ 6.02 (s, 8 *m*-Ar*H*), 3.8 (br s, 24 *o*-Ar*Me*), 2.72 (s, 12 *p*-Ar*Me*). A small amount of carbene dissociation was observed in solution resulting in the observation of small amounts of the dimeric species, [(^{Cl}IMes)FeCl(μ-Cl)]$_2$, and free ^{Cl}IMes. Spectrum provided in section 1.2.

(IPr)Fe(CH$_2$TMS)$_2$. A flask was charged with 382 mg (381 μmol) of [(IPr)FeCl(μ-Cl)]$_2$ and 5 mL of THF. The resulting colorless solution was chilled to 77 K. To the thawing solution was added 1.49 mL (1.48 mmol) of TMSCH$_2$MgCl as a 1.0 M solution in Et$_2$O resulting in a color change to yellow. The yellow solution was allowed to warm to ambient temperature and stir for 20 h. All volatiles were removed in vacuo and the remaining residue was extracted into 5 mL of pentane and filtered. The resulting yellow solution was chilled to −30° C for 20 h during which time the desired compound precipitated as 259 mg (56%) of yellow crystals. Crystals suitable for X-ray diffraction were grown by slow cooling of a saturated pentane solution of the complex at −30 ºC. The ^{1}H NMR spectrum of the compound matched that reported previously.[3] ^{1}H NMR: δ 10.00 (s, 2 carbene-C*H*), 6.42 (s, 4 *m*-Ar*H*), 5.76 (s, 2 *p*-Ar*H*), 2.3 (br s, 12 CH*Me*$_2$), −3.02 (s, 12 CH*Me*$_2$), −9.40 (s, 18 Si*Me*$_3$), −13.3 (v br s, 4 C*H*Me$_2$).

(SIPr)Fe(CH$_2$TMS)$_2$. A flask was charged with 130 mg (333 μmol) of SIPr, 78.3 mg (333 μmol) of FeCl$_2$(THF)$_{1.5}$, and 10 mL of THF. The resulting colorless solution was stirred for one hour and then chilled to 77 K. To the thawing solution was added 1.33 mL (1.33 mmol) of

[1] Arduengo, A. J., III; Krafczyk, R.; Schmutzler, R.; Craig, H. A.; Goerlich, J. R.; Marshall, W. J.; Unverzagt, M. *Tetrahedron* **1999**, *55*, 14523-14534.
[2] Przyojski, J. A.; Arman, H. D.; Tonzetich, Z. J. *Organometallics* **2012**, *31*, 3264-3271.
[3] Danopoulos, A. A.; Braunstein, P.; Wesolek, M.; Monakhov, K. Y.; Rabu, P.; Robert, V. *Organometallics* **2012**, *31*, 4102-4105.

TMSCH$_2$MgCl as a 1.0 M solution in Et$_2$O resulting in a color change to yellow. The yellow solution was allowed to stir for 2.5 h at ambient temperature. All volatiles were removed in vacuo and the remaining yellow residue was dissolved in 5 mL of warm toluene and filtered. The resulting solution was chilled to −30° C for 20 h during which time the desired compound precipitated as 122 mg (59%) of large yellow cubic crystals. The ^{1}H NMR spectrum of the compound matched that reported previously.[3] ^{1}H NMR: δ 16.18 (s, 4 carbene-C*H*$_2$), 6.86 (s, 2 *p*-Ar*H*), 5.92 (s, 4 *m*-Ar*H*), −1.7 (br s, 12 CH*Me*$_2$), −2.78 (s, 12 CH*Me*$_2$), −9.18 (s, 18 Si*Me*$_3$), −19.8 (v br s, 4 C*H*Me$_2$).

(^{Cl}IPr)Fe(CH$_2$TMS)$_2$. A flask was charged with 200 mg (172 μmol) of [(^{Cl}IPr)FeCl(μ-Cl)]$_2$ and 15 mL of Et$_2$O. The resulting suspension was chilled to 77 K. To the thawing suspension was added 686 μL (686 μmol) of TMSCH$_2$MgCl as a 1.0 M solution in Et$_2$O resulting in a color change to yellow. The mixture was allowed to stir for 2 h at ambient temperature. The resulting yellow suspension was filtered through a glass frit. All volatiles were removed in vacuo and the remaining residue was extracted into 5 mL of warm toluene. The toluene solution was filtered and chilled to −30° C for 20 h during which time the desired compound precipitated as 38.0 mg (16%) of yellow crystals. ^{1}H NMR: δ 18.78 (s, 4 *p*-Ar*H*), 18.01 (s, 2 *p*-Ar*H*), −3.33 (s, 12 CH*Me*$_2$), −6.3 (br s, 12 CH*Me*$_2$), −11.83 (s, 18 Si*Me*$_3$), −27.4 (v br s, 4 C*H*Me$_2$). Spectrum provided in section 1.2.

1.2 NMR Spectra

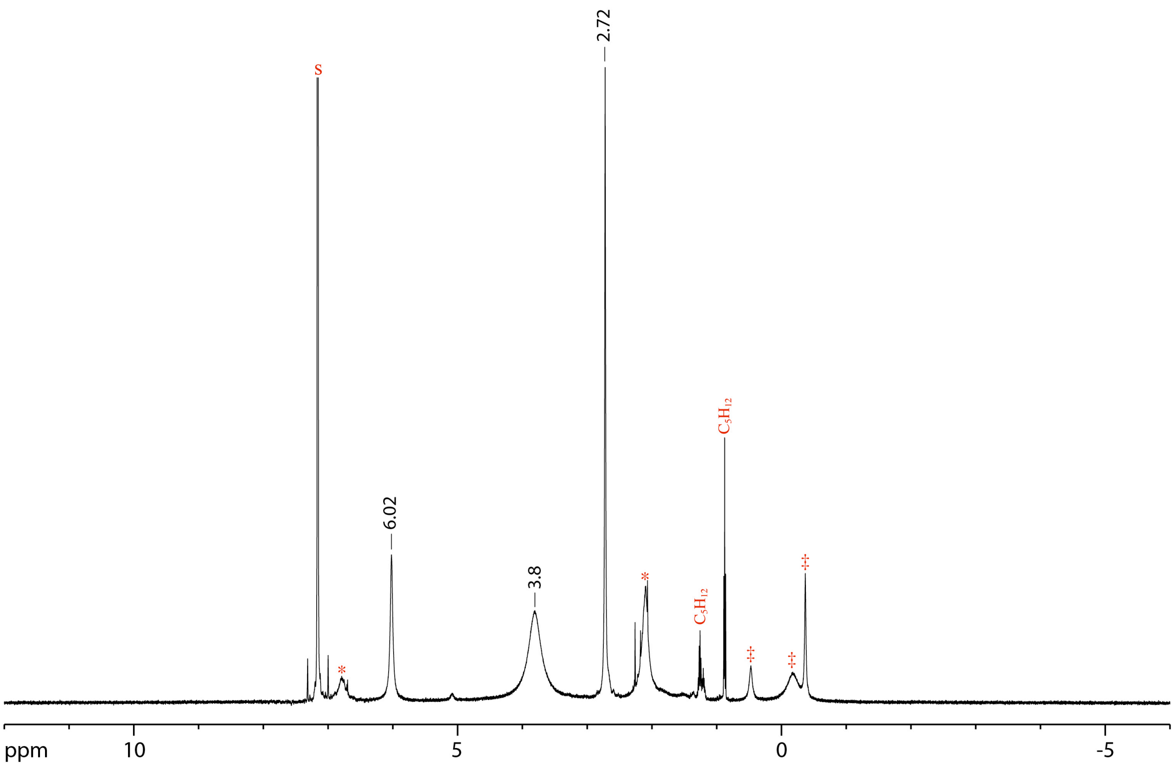

Figure S1. 500 MHz ^{1}H NMR spectrum of (^{Cl}IMes)$_2$FeCl$_2$ in benzene-d_6. Symbols denote residual ^{1}H peak of benzene-d_6 (s), [(^{Cl}IMes)FeCl(μ-Cl)]$_2$ (‡), and free ^{Cl}IMes (*).

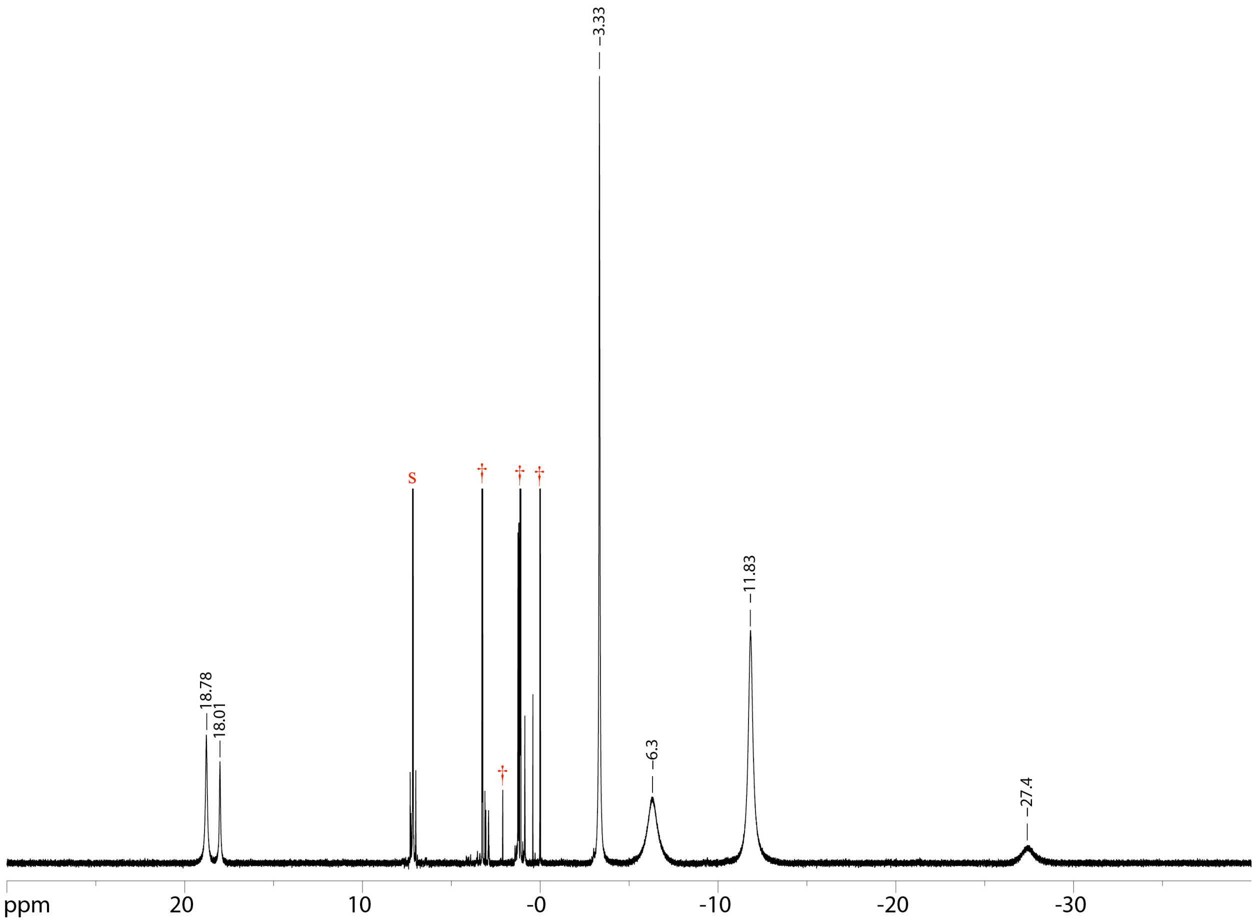

Figure S2. 500 MHz ^{1}H NMR spectrum of (^{Cl}IPr)Fe(CH_2TMS)$_2$ in benzene-d_6. Symbols denote residual ^{1}H peak of benzene-d_6 (s), and small amounts of pentane and toluene from crystallization (†).

2. Supplementary Data

2.1. Mossbauer Spectra

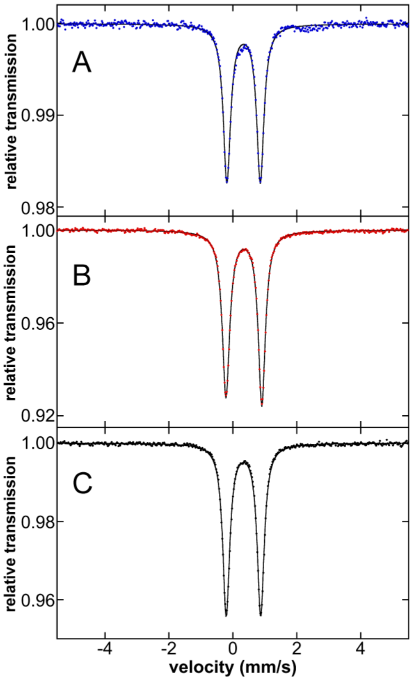

Figure S3. 80 K Mössbauer spectra of (A) (IPr)Fe(CH_2TMS)$_2$, (B) (SIPr)Fe(CH_2TMS)$_2$ and (C) (^{Cl}IPr)Fe(CH_2TMS)$_2$.

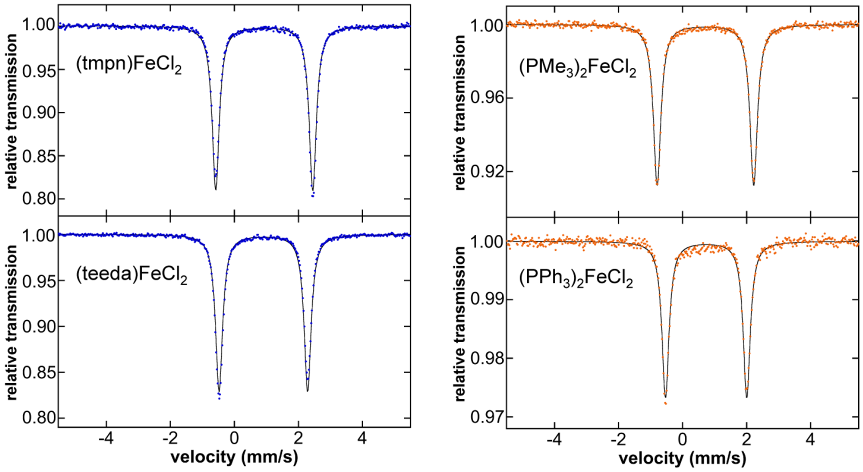

Figure S4. 80 K Mössbauer Spectra of Diamine-$FeCl_2$ and Phosphine-$FeCl_2$ Complexes. Data (dots) and total fit (black lines) are shown for each spectrum. The Mössbauer parameters for each complex were found to be the following: (a) (tmpn)$FeCl_2$, δ = 0.93 mm/s and ΔE_Q = 3.03 mm/s, (b) (teeda)$FeCl_2$, δ = 0.91 mm/s and ΔE_Q = 2.78 mm/s, (c) $(PMe_3)_2FeCl_2$, δ = 0.71 mm/s and ΔE_Q = 3.02 mm/s, and (d) $(PPh_3)_2FeCl_2$, δ = 0.74 mm/s and ΔE_Q = 2.54 mm/s.

2.2. UV-Vis MCD Spectra

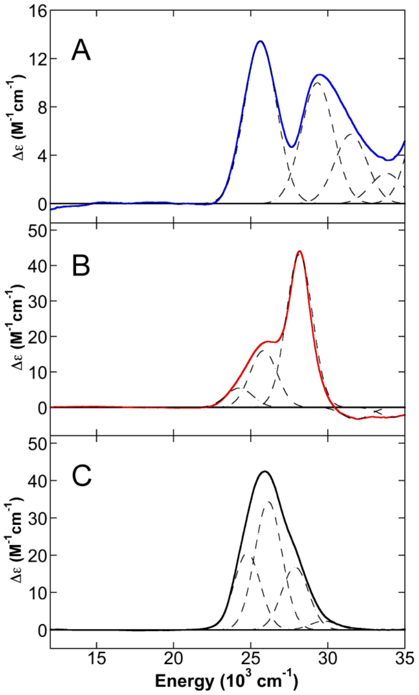

Figure S5. UV-Vis MCD Data of (A) (IPr)Fe(CH_2TMS)$_2$, (B) (SIPr)Fe(CH_2TMS)$_2$ and (C) (^{Cl}IPr)Fe(CH_2TMS)$_2$.

3. DFT Studies

3.1. MO Energy Level Diagrams

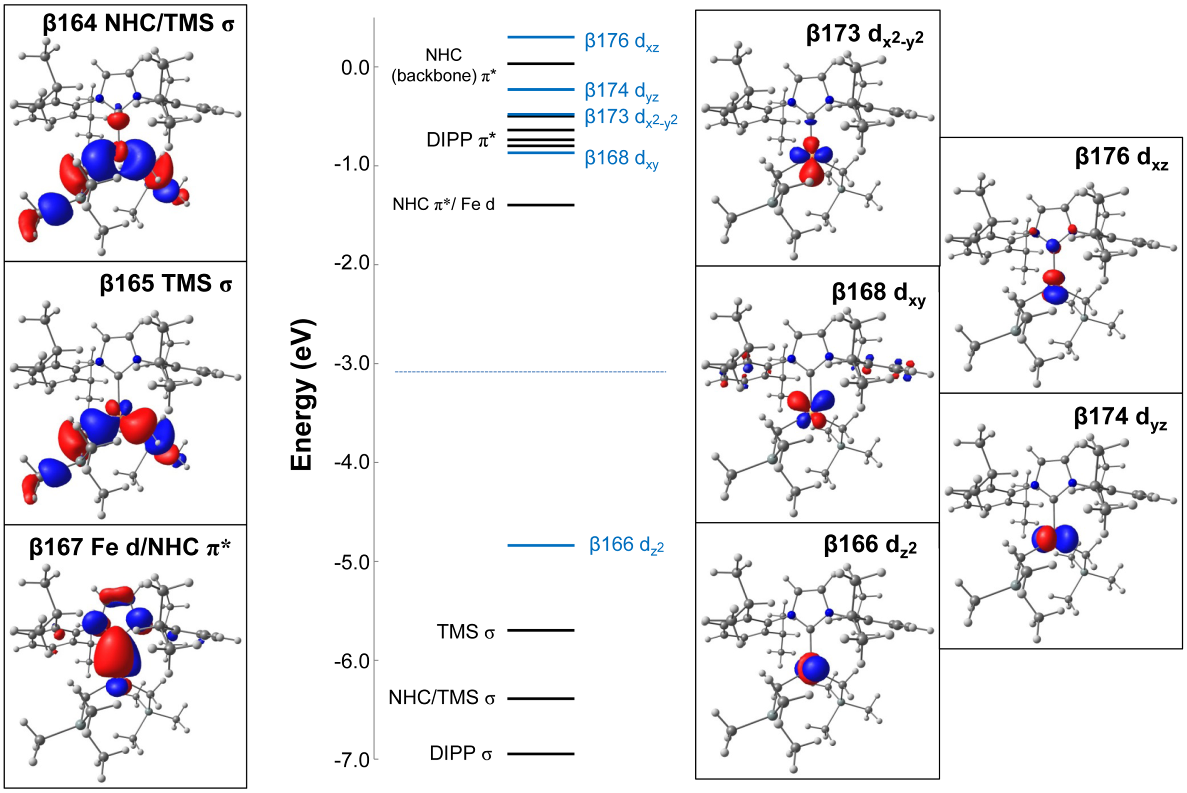

Figure S6. Calculated Molecular Orbital Energy Diagram of (IPr)Fe(CH_2TMS)$_2$.

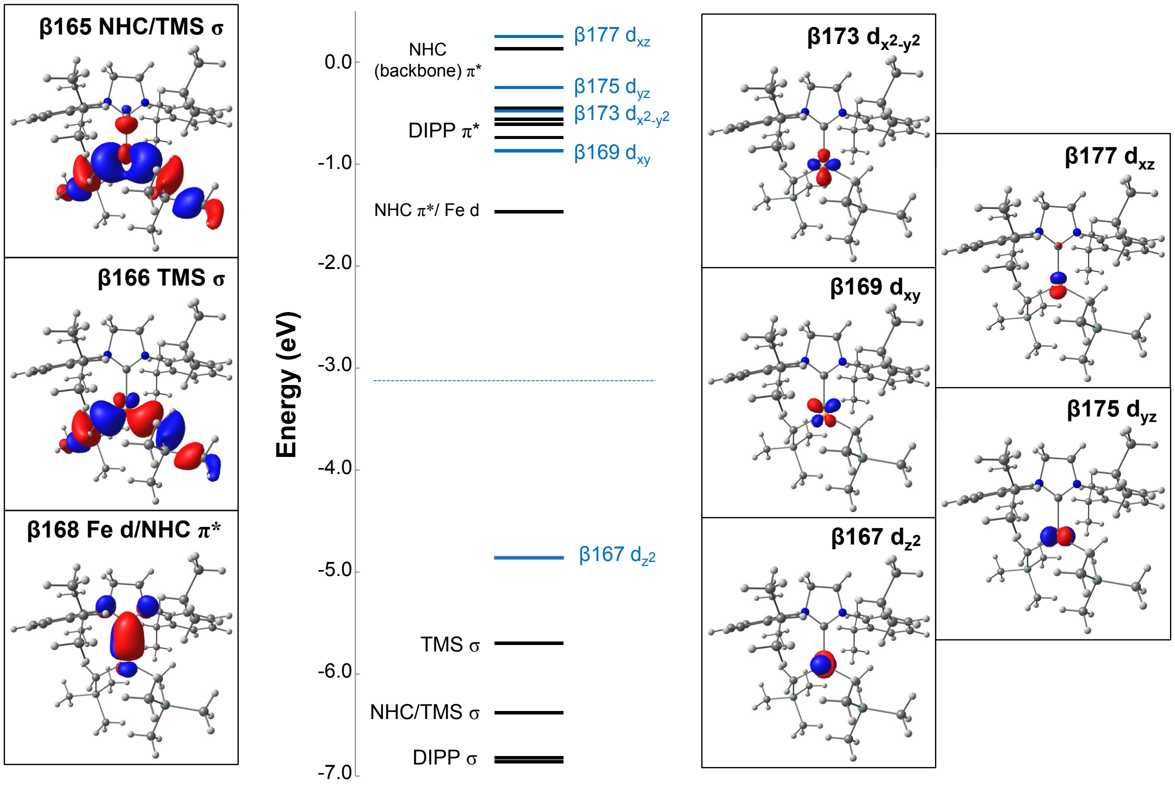

Figure S7. Calculated Molecular Orbital Energy Diagram of (SIPr)Fe(CH_2TMS)$_2$.

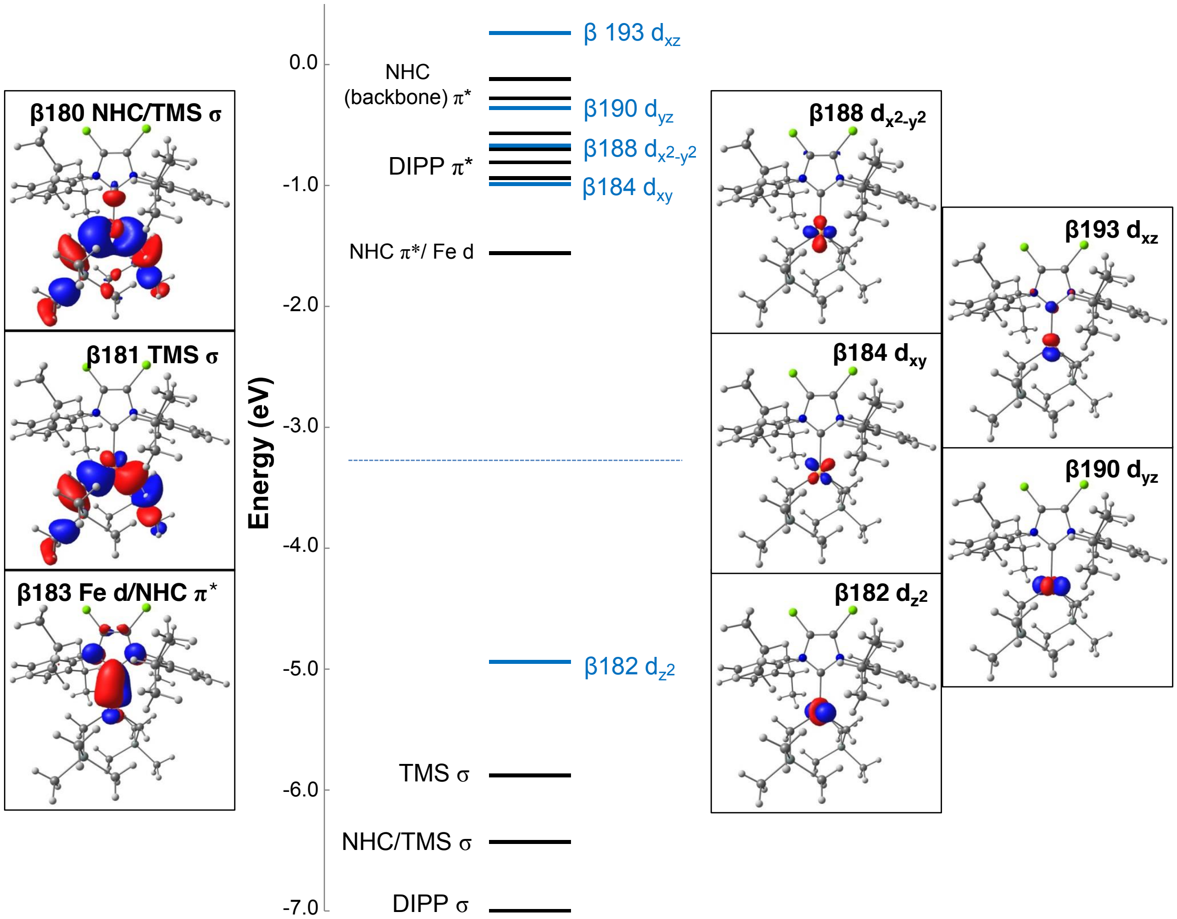

Figure S8. Calculated Molecular Orbital Energy Diagram of (^{Cl}IPr)Fe(CH_2TMS)$_2$.

3.2. TD-DFT

$(IMes)_2FeCl_2$. TD-DFT calculations were used to assign the observed transitions in the MCD spectra of $(IMes)_2FeCl_2$ (see Fig 1B and 1C). Two MCD d-d transitions are observed experimentally at ~5440 cm^{-1} and 6520 cm^{-1} and are assigned as Fe d_{x2-y2}/Cl p→ Fe d_{yz}/Mes π* and Fe d_{x2-y2} → Fe d/NHC σ* transitions, respectively. In general, the charge transfer (CT) transitions are quite mixed, involving multiple NHC and Fe orbitals. The lowest energy CT transition observed experimentally at ~30450 cm^{-1} is assigned as a mixed metal-to-ligand charge transfer (MLCT)/ ligand-to-metal charge transfer (LMCT) transition with contributions of Cl p/ Mes π → Fe d_{z2}/IMes π* andFe d_{x2-y2}/Cl p → Mes π*) to the overall intensity. The transition at ~31890 cm^{-1} is assigned as a Cl p/Mes π → Fe dz^2/IMes π* LMCT transition. The highest energy CT transition at ~33960 cm^{-1} is also mixed and gains intensity from Cl p/IMes π/Mes π → Fe d_{z2}/IMes π* and Fe d_{x2-y2}/Cl p → Mes π* transitions.

$(IPr)Fe(CH_2TMS)_2$. TD-DFT calculations were used to assign the observed transitions in the MCD spectra (see Figure 4A and Figure S3A) of $(IPr)Fe(CH_2TMS)_2$. Two MCD d-d transitions are observed at ~6820 cm^{-1} and ~9360 cm^{-1}, which correlate well to the energy of the calculated transitions and are assigned as Fe d_{z2} → Fe d_{xy}/d_{xz}/π*(NHC) and Fe d_{z2} → Fe d_{x2-y2}/σ*(NHC) transitions, respectively. The lowest energy CT transition observed experimentally at ~25640 cm^{-1} is assigned as a mixed MLCT/LMCT transition with contributions of Fe d_{z2} → π*(NHC)/Fe d_{xz} and σ(TMS)/Fe d_{xy} → π*(IPr)/σ*(NHC)/Fe d_{xz}/Fe d_{xy}/Fe d_{x2-y2} to the overall intensity (where TMS = $(CH_2TMS)_2$). Similarly, the observed transition at ~29330 cm^{-1} is assigned as a mixed MLCT/LMCT transition with contributions of σ(NHC)/σ(TMS) → π*(NHC)/Fe d_{xz}, Fe d_{z2} → π*(NHC)/Fe d_{xz}, and Fe d_{xy}/σ(TMS) → π*(DIPP/NHC) to the intensity (where DIPP = 2,6-diisopropylphenyl). The two highest CT transitions are both mixed LMCT transitions. The observed transition at ~31500 cm^{-1} is assigned as a σ(NHC)/σ(TMS) → Fe d_{xy} LMCT transition while the highest energy transition observed at ~33800 cm^{-1} is assigned as a mixed σ(NHC)/σ(TMS) → Fe d_{x2-y2}/Fed_{yz}/σ*(NHC) and π*(NHC with backbone contributions) → π*(NHC)/Fe d_{xz} transition. In general, the calculated energies of these transitions correlate well with the experimentally observed transitions.

$(SIPr)Fe(CH_2TMS)_2$. TD-DFT calculations were also used to assign the observed transitions in the MCD spectra (see Figure 4B and Figure S3B) of $(SIPr)Fe(CH_2TMS)_2$. Two MCD d-d transitions are observed at ~6350 cm^{-1} and ~9110 cm^{-1}, which correlate well to the energy of the calculated transitions and are assigned as Fe d_{z2} → Fe d_{xy} and Fe d_{z2} → Fe d_{x2-y2}/σ*(NHC) transitions, respectively. The lowest energy CT transition observed experimentally at ~24260 cm^{-1} is assigned as a mixed Fe d_{z2}/σ(TMS) → π*(NHC)/Fe d_{xz} transition. The experimentally observed transition at ~25840 cm^{-1} is assigned as a mixed LMCT transition with contributions of σ(TMS)/Fe dxy → Fe d_{x2-y2}/Fe d_{xy}/σ*(NHC) to the intensity. The next highest energy CT transition observed at ~28160 cm^{-1} is a mixed MLCT/LMCT transition, gaining intensity from Fe d_{z2}/σ(TMS) → π*(NHC/DIPP) and σ(NHC)/σ(TMS) → π*(NHC)/Fe d_{xz} transitions. The observed transition at ~32070 cm^{-1} is assigned as a σ(NHC)/σ(TMS) → Fe d_{xy} LMCT transition. The highest energy transition observed at ~34230 cm^{-1} is assigned as a mixed MLCT/LMCT transition, gaining intensity from Fe d_{z2}/σ(TMS) → π*(NHC with backbone contributions) and σ(TMS)/σ(NHC) → Fe d_{yz} transitions. In general, the calculated energies of these transitions

correlate well with the experimentally observed transitions.

(^{Cl}IPr)Fe(CH$_2$TMS)$_2$. TD-DFT calculations were utilized to assign the observed transitions in the MCD spectra (see Figure 4C and Figure S3C) of (^{Cl}IPr)Fe(CH$_2$TMS)$_2$. Two MCD d-d transitions are observed at ~6520 cm^{-1} and ~9100 cm^{-1}, which correlate well to the energy of the calculated transitions and are assigned as Fe d_{z2} → Fe d_{xy}/d_{xz}/π*(NHC) and Fe d_{z2} → Fe d_{x2-y2}/σ*(NHC) transitions, respectively. The lowest energy CT transition observed experimentally at ~24770 cm^{-1} is assigned as a mixed MLCT transition with contributions of Fe d_{z2} → π*(NHC)/Fe d_{xz}/ Fe d_{xy} and σ(TMS)/Fe d_{xy} → π*(DIPP/NHC) to the overall intensity. The experimentally observed transition at ~26150 cm^{-1} is assigned as a mixed MLCT/LMCT transition with contributions of σ(TMS) → Fe d_{x2-y2}/σ*(NHC) and Fe d_{xy}/σ(TMS) → π*(DIPP/NHC) to the intensity. Similarly, the two highest CT transitions are both mixed MLCT/LMCT transitions. The observed transition at ~27880 cm^{-1} is assigned as a σ(TMS) → Fe d_{xy} LMCT transition mixed with a σ(TMS)/Fe d_{xy} → π*(DIPP) MLCT transition. The highest energy transition observed at ~29750 cm^{-1} is assigned as a mixed σ(TMS)/Fe d_{xy} → π*(NHC)/DIPP, σ*(NHC/TMS)/Fe d_{x2-y2}) → π*(NHC with backbone contributions), and σ(TMS)/σ(NHC) → π*(NHC)/Fe d_{xz} transition. In general, the calculated energies of these transitions correlate well with the experimentally observed transitions.

3.3. Optimized Geometry Coordinates

Table S2. $(IMes)_2FeCl_2$ optimized with B3LYP/TZVP (solvent model)

26	6.072941000	7.961984000	4.574469000
17	4.671788000	6.856036000	6.077902000
17	6.668096000	6.430768000	2.916091000
7	9.127843000	9.035809000	4.739884000
7	8.694965000	7.496080000	6.155890000
7	3.712111000	9.222373000	3.014896000
7	4.123848000	10.546008000	4.640622000
6	8.090195000	8.326018000	5.264215000
6	10.344759000	8.646065000	5.285508000
1	11.271490000	9.104217000	4.991152000
6	10.069792000	7.674919000	6.181105000
1	10.706580000	7.100981000	6.829321000
6	9.019952000	10.201081000	3.905617000
6	9.008858000	11.445150000	4.545791000
6	8.991710000	12.587684000	3.752678000
1	8.984755000	13.558547000	4.233703000
6	8.968613000	12.511399000	2.361692000
6	8.997453000	11.254065000	1.764476000
1	9.003118000	11.178726000	0.683262000
6	9.046556000	10.078872000	2.515390000
6	9.030004000	11.541632000	6.046257000
1	9.023096000	12.582095000	6.363086000
1	9.916961000	11.061882000	6.464395000
1	8.160122000	11.049532000	6.482148000
6	8.877273000	13.759196000	1.525983000
1	9.354972000	13.624901000	0.554813000
1	9.345916000	14.608131000	2.025495000
1	7.831243000	14.022849000	1.342046000
6	9.165460000	8.740933000	1.845845000
1	8.328349000	8.084975000	2.088327000
1	10.071437000	8.223231000	2.170298000
1	9.211133000	8.859362000	0.763793000
6	8.011196000	6.597223000	7.046575000
6	7.392544000	7.131017000	8.178199000
6	6.751803000	6.250124000	9.042815000
1	6.253534000	6.647149000	9.919775000
6	6.710421000	4.880937000	8.793034000
6	7.349010000	4.390513000	7.657075000
1	7.321464000	3.327363000	7.447867000
6	8.009087000	5.231175000	6.764298000
6	7.373520000	8.611895000	8.436223000
1	8.365085000	9.056581000	8.333460000

1	7.005220000	8.820780000	9.440113000
1	6.716893000	9.118534000	7.726601000
6	5.948441000	3.957569000	9.705154000
1	5.956844000	4.317639000	10.735116000
1	6.363482000	2.948873000	9.689486000
1	4.903726000	3.889169000	9.388835000
6	8.660509000	4.687031000	5.523994000
1	8.537334000	3.605784000	5.474087000
1	9.730144000	4.907636000	5.496739000
1	8.210885000	5.125484000	4.630584000
6	4.628195000	9.459971000	3.991323000
6	2.662321000	10.127399000	3.057785000
1	1.847983000	10.087401000	2.357523000
6	2.920075000	10.962636000	4.086000000
1	2.382298000	11.811146000	4.468458000
6	3.841050000	8.227013000	1.984309000
6	4.692852000	8.495810000	0.911645000
6	4.796734000	7.536996000	-0.090163000
1	5.463001000	7.722230000	-0.924952000
6	4.087391000	6.339732000	-0.032436000
6	3.242533000	6.117011000	1.051446000
1	2.688180000	5.187664000	1.112483000
6	3.101842000	7.048123000	2.077717000
6	5.506483000	9.758740000	0.856276000
1	5.998413000	9.855644000	-0.110942000
1	6.279444000	9.755184000	1.626274000
1	4.892832000	10.646550000	1.021136000
6	4.269754000	5.288334000	-1.093850000
1	4.493582000	5.735188000	-2.063777000
1	3.378693000	4.667775000	-1.197751000
1	5.102675000	4.628389000	-0.835131000
6	2.217956000	6.769400000	3.260460000
1	1.697633000	5.821121000	3.130612000
1	1.469111000	7.551539000	3.403127000
1	2.810479000	6.714099000	4.176463000
6	4.817797000	11.339516000	5.617874000
6	5.569788000	12.419739000	5.143709000
6	6.189185000	13.243836000	6.077538000
1	6.773612000	14.085817000	5.726053000
6	6.086466000	13.003401000	7.446181000
6	5.309223000	11.931535000	7.876566000
1	5.197522000	11.747978000	8.938919000
6	4.642593000	11.094534000	6.980744000
6	5.691840000	12.688872000	3.669574000
1	6.194808000	11.865406000	3.161968000
1	6.268122000	13.594259000	3.493229000

1	4.712472000	12.808705000	3.202841000
6	6.826141000	13.864883000	8.433628000
1	6.943741000	14.884117000	8.063061000
1	7.828167000	13.465277000	8.617749000
1	6.309895000	13.905265000	9.393445000
6	3.737728000	10.003014000	7.473339000
1	2.712249000	10.166988000	7.132444000
1	3.731741000	9.975827000	8.562491000
1	4.040871000	9.022960000	7.103432000

Table S3. $(PMe_3)_2FeCl_2$ optimized with B3LYP/TZVP (solvent model)

26	-1.564511000	7.443517000	6.411266000
17	-1.406181000	9.630604000	5.793118000
17	-0.531826000	5.480586000	5.881907000
15	-1.330781000	7.569775000	8.842268000
6	0.278905000	8.281454000	9.389095000
1	0.348540000	8.326733000	10.477102000
1	0.379938000	9.283946000	8.973924000
1	1.088859000	7.665215000	8.998962000
6	-1.405830000	5.954666000	9.726419000
1	-1.236345000	6.079050000	10.797010000
1	-0.650591000	5.290066000	9.308344000
1	-2.383230000	5.500960000	9.564976000
6	-2.570746000	8.608444000	9.729525000
1	-2.353780000	8.664769000	10.797344000
1	-3.565422000	8.184658000	9.589176000
1	-2.560524000	9.611463000	9.303586000
15	-3.928447000	6.913262000	6.052017000
6	-4.651474000	5.570693000	7.090437000
1	-5.681279000	5.355077000	6.801493000
1	-4.631113000	5.870590000	8.138271000
1	-4.046895000	4.671158000	6.977111000
6	-5.088605000	8.324262000	6.296251000
1	-6.115601000	8.041512000	6.060292000
1	-4.776641000	9.148261000	5.655493000
1	-5.034157000	8.662127000	7.331038000
6	-4.292679000	6.351592000	4.334920000
1	-5.351132000	6.120356000	4.205336000
1	-3.695134000	5.466044000	4.119757000
1	-4.004316000	7.137162000	3.636563000

Table S4. $(PPh_3)_2FeCl_2$ optimized with B3LYP/TZVP (solvent model)

26	0.000005000	-0.000036000	1.568700000

17	0.008548000	-2.055619000	2.533093000
15	-2.033875000	-0.006109000	0.146339000
6	-3.542085000	-0.115263000	1.189663000
6	-4.667598000	-0.838493000	0.793166000
1	-4.662485000	-1.376064000	-0.145434000
6	-5.795339000	-0.872714000	1.605568000
1	-6.664255000	-1.438888000	1.294240000
6	-5.806235000	-0.184227000	2.814700000
1	-6.684366000	-0.214496000	3.447430000
6	-4.685111000	0.537693000	3.212731000
1	-4.686444000	1.069831000	4.155476000
6	-3.552887000	0.569132000	2.407581000
1	-2.681313000	1.129034000	2.723607000
6	-2.318095000	1.479162000	-0.897421000
6	-1.278729000	1.924864000	-1.717095000
1	-0.329238000	1.409573000	-1.724720000
6	-1.451994000	3.038381000	-2.528427000
1	-0.635109000	3.372825000	-3.153960000
6	-2.661276000	3.727101000	-2.516604000
1	-2.794636000	4.601231000	-3.141468000
6	-3.695982000	3.294021000	-1.694168000
1	-4.637108000	3.829198000	-1.677199000
6	-3.528381000	2.173110000	-0.887711000
1	-4.337739000	1.843580000	-0.251217000
6	-2.202495000	-1.420949000	-1.016477000
6	-2.535782000	-1.251849000	-2.360232000
1	-2.726916000	-0.262294000	-2.751828000
6	-2.618147000	-2.357001000	-3.202687000
1	-2.874284000	-2.217196000	-4.245449000
6	-2.378855000	-3.633705000	-2.706754000
1	-2.443803000	-4.492005000	-3.363693000
6	-2.048859000	-3.805089000	-1.364850000
1	-1.849647000	-4.795152000	-0.975182000
6	-1.953275000	-2.705685000	-0.523087000
1	-1.664469000	-2.841528000	0.511332000
17	-0.008558000	2.055503000	2.533183000
15	2.033892000	0.006106000	0.146345000
6	3.542116000	0.115200000	1.189650000
6	4.667640000	0.838416000	0.793160000
1	4.662525000	1.376021000	-0.145420000
6	5.795397000	0.872579000	1.605545000
1	6.664322000	1.438743000	1.294221000
6	5.806298000	0.184047000	2.814650000
1	6.684441000	0.214270000	3.447364000
6	4.685162000	-0.537860000	3.212675000
1	4.686500000	-1.070032000	4.155400000

6	3.552923000	-0.569241000	2.407544000
1	2.681341000	-1.129134000	2.723564000
6	2.318094000	-1.479109000	-0.897498000
6	1.278716000	-1.924752000	-1.717189000
1	0.329232000	-1.409448000	-1.724776000
6	1.451959000	-3.038226000	-2.528584000
1	0.635066000	-3.372623000	-3.154130000
6	2.661234000	-3.726962000	-2.516807000
1	2.794576000	-4.601059000	-3.141721000
6	3.695951000	-3.293942000	-1.694354000
1	4.637069000	-3.829134000	-1.677421000
6	3.528371000	-2.173073000	-0.887834000
1	4.337738000	-1.843588000	-0.251328000
6	2.202491000	1.421004000	-1.016402000
6	2.535742000	1.251972000	-2.360174000
1	2.726867000	0.262436000	-2.751825000
6	2.618084000	2.357166000	-3.202576000
1	2.874193000	2.217414000	-4.245352000
6	2.378801000	3.633844000	-2.706573000
1	2.443729000	4.492177000	-3.363471000
6	2.048842000	3.805161000	-1.364651000
1	1.849638000	4.795204000	-0.974927000
6	1.953282000	2.705715000	-0.522941000
1	1.664503000	2.841505000	0.511493000

Table S5. (tmpn)$FeCl_2$ optimized with uB3LYP/TZVP (solvent model)

26	4.782640000	5.807746000	-3.173009000
17	4.782658000	7.124101000	-1.291832000
17	4.782616000	6.141660000	-5.423395000
7	3.143861000	4.431695000	-2.719563000
6	4.782624000	2.832250000	-1.592425000
1	4.782609000	2.165843000	-2.455737000
1	4.782624000	2.171049000	-0.722200000
6	3.484501000	3.632945000	-1.514970000
1	2.653550000	2.944998000	-1.308909000
1	3.545539000	4.332502000	-0.680552000
6	1.949604000	5.252551000	-2.421657000
1	1.699584000	5.851053000	-3.296369000
1	2.168259000	5.919332000	-1.591026000
1	1.093164000	4.616450000	-2.168025000
6	2.840045000	3.559371000	-3.870826000
1	3.708952000	2.968096000	-4.144543000
1	2.575822000	4.177134000	-4.725452000
1	2.009276000	2.884145000	-3.633680000
6	6.080764000	3.632921000	-1.514991000

7	6.421407000	4.431680000	-2.719580000
6	7.615669000	5.252524000	-2.421666000
1	7.865690000	5.851035000	-3.296371000
1	7.397017000	5.919295000	-1.591026000
1	8.472104000	4.616414000	-2.168044000
6	6.725220000	3.559374000	-3.870859000
1	5.856310000	2.968112000	-4.144591000
1	6.989451000	4.177152000	-4.725472000
1	7.555983000	2.884137000	-3.633722000
1	6.911707000	2.944959000	-1.308942000
1	6.019749000	4.332474000	-0.680568000

Table S6. (teeda)$FeCl_2$ optimized with uB3LYP/TZVP (solvent model)

26	0.276099000	0.150892000	5.157647000
17	2.513173000	0.578430000	4.981016000
17	-0.874192000	-1.707582000	5.763028000
7	-0.444064000	1.930725000	6.240113000
6	-0.589857000	2.905316000	5.134934000
1	0.418211000	3.168149000	4.812727000
1	-1.067733000	3.830906000	5.472351000
6	-1.374976000	2.331846000	3.965818000
1	-2.386445000	2.083948000	4.283548000
1	-1.467630000	3.100118000	3.192224000
7	-0.745008000	1.102805000	3.419385000
6	-1.739353000	1.596884000	6.897083000
1	-1.544923000	0.771270000	7.577042000
1	-2.401154000	1.191806000	6.134182000
6	-2.432218000	2.738989000	7.633854000
1	-3.372020000	2.371182000	8.048621000
1	-2.669418000	3.576204000	6.975939000
1	-1.831981000	3.115990000	8.462278000
6	0.566195000	2.436958000	7.208634000
1	0.249770000	3.413353000	7.592520000
1	1.482750000	2.587923000	6.641242000
6	0.844111000	1.483445000	8.358438000
1	1.692098000	1.858245000	8.933077000
1	1.104769000	0.490748000	7.990154000
1	-0.001982000	1.389250000	9.039443000
6	0.270675000	1.476682000	2.392760000
1	1.020678000	2.085153000	2.895976000
1	-0.204494000	2.106919000	1.632548000
6	0.956438000	0.293323000	1.731409000
1	1.755336000	0.665353000	1.088381000
1	0.274822000	-0.287938000	1.110259000
1	1.411600000	-0.363662000	2.471293000

6	-1.776475000	0.168660000	2.887388000
1	-2.436764000	-0.072032000	3.719686000
1	-1.273706000	-0.760561000	2.629099000
6	-2.585854000	0.681404000	1.700362000
1	-3.330219000	-0.068448000	1.427957000
1	-1.962762000	0.860458000	0.823476000
1	-3.118497000	1.604887000	1.934111000

Table S7. (IPr)Fe(CH_2TMS)$_2$ optimized with uB3LYP/TZVP (solvent model)

26	2.179811000	0.256460000	13.530972000
14	-0.405155000	-1.856934000	12.602646000
6	0.974686000	-0.693837000	12.103198000
1	1.677683000	-1.274038000	11.485722000
1	0.559609000	0.074168000	11.437495000
6	-1.440375000	-2.433714000	11.101476000
1	-1.895084000	-1.582643000	10.586793000
1	-0.813198000	-2.959003000	10.375744000
1	-2.245245000	-3.111889000	11.401047000
6	-1.628667000	-1.046230000	13.822992000
1	-2.062139000	-0.128898000	13.417281000
1	-2.453365000	-1.727008000	14.052803000
1	-1.137612000	-0.792970000	14.765496000
6	0.258495000	-3.427363000	13.448941000
1	0.924509000	-3.985090000	12.786156000
1	0.821783000	-3.186305000	14.353219000
1	-0.564212000	-4.090739000	13.730712000
14	4.765469000	-1.856587000	14.458127000
6	3.384907000	-0.694638000	14.958230000
1	2.682032000	-1.275774000	15.574961000
1	3.799399000	0.072980000	15.624738000
6	5.801452000	-2.433056000	15.958888000
1	6.255619000	-1.581801000	16.473746000
1	5.174838000	-2.959061000	16.684588000
1	6.606778000	-3.110516000	15.658925000
6	5.988156000	-1.044761000	13.237689000
1	6.421158000	-0.127256000	13.643511000
1	6.813218000	-1.724968000	13.007491000
1	5.496689000	-0.791575000	12.295378000
6	4.102721000	-3.427237000	13.611523000
1	3.437137000	-3.985555000	14.274244000
1	3.539192000	-3.186333000	12.707354000
1	4.925831000	-4.090010000	13.329509000
7	1.980158000	3.289623000	12.478563000
6	2.179920000	2.437974000	13.531636000
6	1.693748000	2.919070000	11.109563000

6	0.363987000	2.993678000	10.660824000
6	2.758202000	2.575215000	10.258161000
6	2.056614000	4.620067000	12.869975000
1	1.927476000	5.430241000	12.175536000
6	4.209677000	2.577710000	10.722012000
1	4.215169000	2.463675000	11.806573000
6	0.115577000	2.689663000	9.321391000
1	-0.898439000	2.732044000	8.945453000
6	-0.790498000	3.415378000	11.561234000
1	-0.430399000	3.424436000	12.590278000
6	2.449977000	2.277273000	8.930198000
1	3.243278000	2.001609000	8.248575000
6	1.144561000	2.332500000	8.464360000
1	0.929485000	2.094770000	7.429611000
6	5.028302000	1.415295000	10.142913000
1	4.539399000	0.455570000	10.316282000
1	5.187371000	1.523136000	9.067947000
1	6.012175000	1.383620000	10.614436000
6	4.881370000	3.924488000	10.393107000
1	4.350577000	4.762152000	10.848811000
1	5.910004000	3.936339000	10.761192000
1	4.907192000	4.090595000	9.313351000
6	-1.264504000	4.840599000	11.221911000
1	-0.453737000	5.567232000	11.298772000
1	-1.658619000	4.888272000	10.204024000
1	-2.059891000	5.148862000	11.904533000
6	-1.970175000	2.432834000	11.503177000
1	-1.648264000	1.413477000	11.713788000
1	-2.724706000	2.712291000	12.241836000
1	-2.453850000	2.438257000	10.524339000
7	2.379720000	3.288918000	14.585279000
6	2.665957000	2.917469000	15.954074000
6	3.995649000	2.991837000	16.403059000
6	1.601393000	2.573038000	16.805110000
6	2.303283000	4.619624000	14.194749000
1	2.432444000	5.429335000	14.889725000
6	0.149975000	2.575701000	16.341072000
1	0.144619000	2.462282000	15.256445000
6	4.243870000	2.687034000	17.742348000
1	5.257829000	2.729241000	18.118462000
6	5.150260000	3.414081000	15.503064000
1	4.790354000	3.423565000	14.473955000
6	1.909436000	2.274301000	18.132935000
1	1.116043000	1.998182000	18.814267000
6	3.214775000	2.329313000	18.599009000
1	3.429703000	2.090972000	19.633649000

6	-0.668632000	1.412886000	16.919401000
1	-0.179629000	0.453297000	16.745561000
1	-0.827861000	1.520107000	17.994405000
1	-1.652436000	1.381397000	16.447721000
6	-0.521873000	3.922227000	16.670683000
1	0.008903000	4.760211000	16.215552000
1	-1.550461000	3.934207000	16.302475000
1	-0.547851000	4.087687000	17.750536000
6	5.624061000	4.839188000	15.843138000
1	4.813247000	5.565785000	15.766421000
1	6.017937000	4.886432000	16.861137000
1	6.419576000	5.147834000	15.160839000
6	6.330023000	2.431624000	15.560893000
1	6.008225000	1.412315000	15.349881000
1	7.084609000	2.711419000	14.822418000
1	6.813596000	2.436734000	16.539784000

Table S8. (SIPr)Fe(CH_2TMS)$_2$ optimized with uB3LYP/TZVP (solvent model)

26	4.410362000	9.522013000	4.482139000
14	1.932355000	11.671435000	3.323747000
7	4.121900000	6.518840000	3.435362000
6	4.409985000	7.320924000	4.481960000
6	4.320649000	5.077687000	3.725031000
1	5.204981000	4.715002000	3.195441000
1	3.462801000	4.494984000	3.394918000
6	3.790595000	6.900159000	2.088904000
6	2.444524000	6.819572000	1.680331000
6	2.139616000	7.129020000	0.354875000
1	1.110746000	7.086183000	0.020098000
6	3.131532000	7.491000000	-0.543984000
1	2.873987000	7.732923000	-1.568041000
6	4.453472000	7.541485000	-0.130269000
1	5.221266000	7.815701000	-0.842612000
6	4.814507000	7.238867000	1.183879000
6	1.323368000	6.398082000	2.621620000
1	1.753585000	6.269632000	3.615333000
6	0.708849000	5.051216000	2.198987000
1	0.218752000	5.129972000	1.225983000
1	-0.043140000	4.731995000	2.924653000
1	1.463026000	4.264976000	2.124289000
6	0.231295000	7.472571000	2.738151000
1	-0.511406000	7.177018000	3.482963000
1	-0.291747000	7.617188000	1.790497000
1	0.651784000	8.431944000	3.037477000
6	6.287369000	7.255811000	1.570369000

1	6.353056000	7.132679000	2.651582000
6	7.046084000	6.083026000	0.922262000
1	7.040963000	6.165937000	-0.167035000
1	6.601434000	5.120181000	1.182496000
1	8.087920000	6.075600000	1.251718000
6	6.964421000	8.591095000	1.223654000
1	6.422559000	9.435171000	1.651445000
1	7.020566000	8.745018000	0.143966000
1	7.984606000	8.609784000	1.612243000
6	3.328468000	10.473035000	2.960536000
1	4.078693000	11.021583000	2.371619000
1	2.945597000	9.689496000	2.294939000
6	0.782762000	11.903172000	1.813637000
1	0.336523000	10.952758000	1.508117000
1	1.339896000	12.293982000	0.957608000
1	-0.031329000	12.601151000	2.031426000
6	0.820026000	11.104108000	4.767975000
1	1.382490000	11.069530000	5.704211000
1	0.397095000	10.110833000	4.598646000
1	-0.014734000	11.796819000	4.909382000
6	2.583877000	13.397598000	3.790181000
1	3.213061000	13.811945000	2.997885000
1	3.179653000	13.369229000	4.704500000
1	1.754227000	14.091651000	3.952340000
7	4.698012000	6.518517000	5.528330000
6	4.499156000	5.077464000	5.238246000
1	3.614800000	4.714690000	5.767735000
1	5.356964000	4.494607000	5.568192000
6	5.029476000	6.899403000	6.874874000
6	6.375606000	6.818716000	7.283227000
6	6.680703000	7.127781000	8.608728000
1	7.709624000	7.084879000	8.943342000
6	5.688911000	7.489475000	9.507839000
1	5.946603000	7.731110000	10.531927000
6	4.366906000	7.540029000	9.094339000
1	3.599212000	7.814002000	9.806883000
6	4.005683000	7.237779000	7.780157000
6	7.496635000	6.397544000	6.341642000
1	7.066237000	6.269187000	5.347996000
6	8.111434000	5.050698000	6.763925000
1	8.601731000	5.129353000	7.736835000
1	8.863309000	4.731701000	6.038042000
1	7.357375000	4.264347000	6.838650000
6	8.588520000	7.472223000	6.225055000
1	9.331151000	7.176867000	5.480095000
1	9.111684000	7.616822000	7.172644000

1	8.167828000	8.431559000	5.925895000
6	2.532758000	7.254704000	7.393913000
1	2.466907000	7.132024000	6.312658000
6	1.774308000	6.081540000	8.041648000
1	1.779583000	6.163998000	9.130979000
1	2.219058000	5.118868000	7.780948000
1	0.732424000	6.074101000	7.712348000
6	1.855585000	8.589742000	7.741320000
1	2.397256000	9.434072000	7.313796000
1	1.799602000	8.743190000	8.821084000
1	0.835331000	8.608464000	7.352912000
14	6.888511000	11.671079000	5.640697000
6	5.492896000	10.472116000	6.003851000
1	4.742880000	11.020005000	6.593649000
1	5.876364000	9.688170000	6.668626000
6	8.037735000	11.903609000	7.150967000
1	8.484333000	10.953435000	7.456716000
1	7.480282000	12.294312000	8.006837000
1	8.851557000	12.601911000	6.933207000
6	8.001299000	11.103822000	4.196803000
1	7.438975000	11.068625000	3.260508000
1	8.424694000	10.110816000	4.366554000
1	8.835740000	11.796890000	4.055260000
6	6.236302000	13.396848000	5.173791000
1	5.606771000	13.811063000	5.965880000
1	5.640707000	13.368000000	4.259369000
1	7.065654000	14.091260000	5.011648000

Table S9. (^{Cl}IPr)Fe(CH_2TMS)$_2$ optimized with uB3LYP/TZVP (solvent model)

26	2.179862000	0.421880000	13.531102000
14	-0.349602000	-1.895617000	12.911004000
7	1.998087000	3.493239000	12.468079000
6	2.179835000	2.655625000	13.531814000
6	1.710861000	3.076244000	11.109614000
6	0.375622000	3.106723000	10.670646000
6	2.777060000	2.698109000	10.278364000
6	2.075047000	4.822309000	12.863827000
6	4.234513000	2.740673000	10.717806000
1	4.263097000	2.971833000	11.782709000
6	0.124068000	2.693754000	9.362345000
1	-0.893354000	2.691909000	8.992640000
6	0.905969000	-0.649941000	12.283543000
1	1.638891000	-1.207237000	11.680559000
1	0.399133000	0.026033000	11.583467000
6	-0.785505000	3.571716000	11.542336000

1	-0.384306000	3.903270000	12.500461000
6	2.463334000	2.298260000	8.978414000
1	3.258465000	1.993971000	8.310221000
6	1.153239000	2.288598000	8.525690000
1	0.933427000	1.969041000	7.514310000
6	4.931954000	1.384718000	10.533154000
1	4.403044000	0.593163000	11.066002000
1	4.991859000	1.100839000	9.480519000
1	5.952069000	1.432550000	10.919622000
6	-0.798241000	-3.169938000	11.558811000
1	-1.202210000	-2.672748000	10.672417000
1	0.084908000	-3.733573000	11.245370000
1	-1.544979000	-3.888268000	11.910448000
6	4.998418000	3.858116000	9.984807000
1	4.532258000	4.832387000	10.142855000
1	6.027911000	3.910568000	10.346503000
1	5.031574000	3.673221000	8.908776000
6	-1.984805000	-1.060933000	13.428020000
1	-2.477454000	-0.588188000	12.574872000
1	-2.676030000	-1.802435000	13.838655000
1	-1.828492000	-0.295442000	14.191183000
6	0.258711000	-2.896476000	14.414458000
1	1.191314000	-3.422836000	14.199203000
1	0.436896000	-2.252903000	15.279110000
1	-0.486070000	-3.644694000	14.700752000
6	-1.519176000	4.770684000	10.916012000
1	-0.837895000	5.596995000	10.710021000
1	-2.005414000	4.493006000	9.978614000
1	-2.294811000	5.130445000	11.595891000
6	-1.769280000	2.429272000	11.837537000
1	-1.272788000	1.590587000	12.322678000
1	-2.566893000	2.781531000	12.495761000
1	-2.234634000	2.059513000	10.921070000
14	4.709938000	-1.895514000	14.149422000
7	2.361480000	3.492637000	14.596042000
6	2.648744000	3.074877000	15.954264000
6	3.983971000	3.105337000	16.393289000
6	1.582593000	2.696077000	16.785271000
6	2.284304000	4.821931000	14.201080000
6	0.125138000	2.738620000	16.345827000
1	0.096531000	2.970164000	15.281005000
6	4.235562000	2.691649000	17.701354000
1	5.252971000	2.689790000	18.071095000
6	3.454127000	-0.650533000	14.777787000
1	2.721406000	-1.208310000	15.380574000
1	3.960944000	0.025136000	15.478167000

6	5.145035000	3.571172000	15.521956000
1	4.743779000	3.903527000	14.564133000
6	1.896352000	2.295571000	18.085013000
1	1.101253000	1.990809000	18.753030000
6	3.206436000	2.285860000	18.537759000
1	3.426274000	1.965796000	19.548974000
6	-0.572120000	1.382505000	16.530015000
1	-0.043004000	0.591162000	15.997055000
1	-0.632136000	1.098349000	17.582569000
1	-1.592187000	1.430276000	16.143414000
6	5.158209000	-3.171219000	15.500433000
1	5.561920000	-2.674930000	16.387448000
1	4.274978000	-3.735193000	15.813028000
1	5.905061000	-3.889165000	15.148254000
6	-0.638918000	3.855698000	17.079214000
1	-0.172923000	4.830096000	16.921454000
1	-1.668436000	3.908096000	16.717581000
1	-0.671994000	3.670457000	18.155188000
6	6.345270000	-1.060121000	13.633919000
1	6.837654000	-0.588453000	14.487818000
1	7.036622000	-1.801104000	13.222558000
1	6.189205000	-0.293668000	12.871669000
6	4.102413000	-2.894918000	12.644671000
1	3.169975000	-3.421927000	12.859058000
1	3.924223000	-2.250472000	11.780667000
1	4.847606000	-3.642499000	12.357787000
6	5.878627000	4.769648000	16.149308000
1	5.197290000	5.595725000	16.356046000
1	6.364914000	4.491179000	17.086447000
1	6.654216000	5.130062000	15.469722000
6	6.128900000	2.429075000	15.225733000
1	5.632505000	1.590822000	14.739747000
1	6.926537000	2.782017000	14.567903000
1	6.594218000	2.058474000	16.141878000
17	2.406588000	6.159257000	15.268935000
17	1.952496000	6.160249000	11.796772000

4. X-ray Crystallography

4.1 Notes on Structure Refinement

(^{Cl}IMes)$_2$FeCl$_2$ and (IPr)Fe(CH$_2$TMS)$_2$. Crystals suitable for X-ray diffraction were mounted in Paratone oil onto a glass fiber and frozen under a nitrogen cold stream maintained by a X-Stream low-temperature apparatus. Diffraction data were collected at 98(2) K using a Rigaku AFC12/Saturn 724 CCD fitted with Mo Kα radiation (λ = 0.71073 Å). Data collection and unit cell refinement were performed using *Crystal Clear* software.[4] Data processing and absorption correction, giving minimum and maximum transmission factors were accomplished with Crystal Clear and ABSCOR, respectively.[5] All structures were solved by direct methods and refined on F^2 using full-matrix, least-squares techniques with SHELXL-97.[6] All non-hydrogen atoms were refined with anisotropic displacement parameters. All carbon bound hydrogen atom positions were determined by geometry and refined by a riding model.

(PMe$_3$)$_2$FeCl$_2$. A crystal was placed onto the tip of an optical fiber and mounted on a Bruker SMART APEX II CCD platform diffractometer for a data collection at 223(2) K.[7] Data collection was carried out using MoKα radiation (graphite monochromator) with a frame time of 60 seconds and a detector distance of 4.04 cm. The structure was solved using SIR2011[8] and refined using SHELXL-2014.[9] A direct-methods solution was calculated which provided most non-hydrogen atoms from the E-map. Full-matrix least squares / difference Fourier cycles were performed which located the remaining non-hydrogen atoms. All non-hydrogen atoms were refined with anisotropic displacement parameters. All hydrogen atoms were placed in ideal positions and refined as riding atoms with relative isotropic displacement parameters.

[4] *Crystal Clear.* Rigaku/MSC Inc.; Rigaku Corporation, The Woodlands, TX, 2005.
[5] *ABSCOR.* Higashi; Rigaku Corporation, Tokyo, Japan, 1995.
[6] Sheldrick, G. M. *Acta Crystallogr., Sect. A* **2008**, *A64*, 112-122.
[7] *APEX2*, version 2013.10-0; Bruker AXS: Madison, WI, 2013.
[8] Burla, M. C.; Caliandro, R.; Camalli, M.; Carrozzini, B.; Cascarano, G. L.; Giacovazzo, C.; Mallamo, M.; Mazzone, A.; Polidori, G.; Spagna, R. *SIR2011: A new package for crystal structure determination and refinement, version 1.0*; Istituto di Cristallografia; Bari, Italy, 2012.
[9] Sheldrick, G. M. *SHELXL-2014/1*; University of Göttingen: Göttingen, Germany, 2014.

4.2 Thermal Ellipsoid Drawings

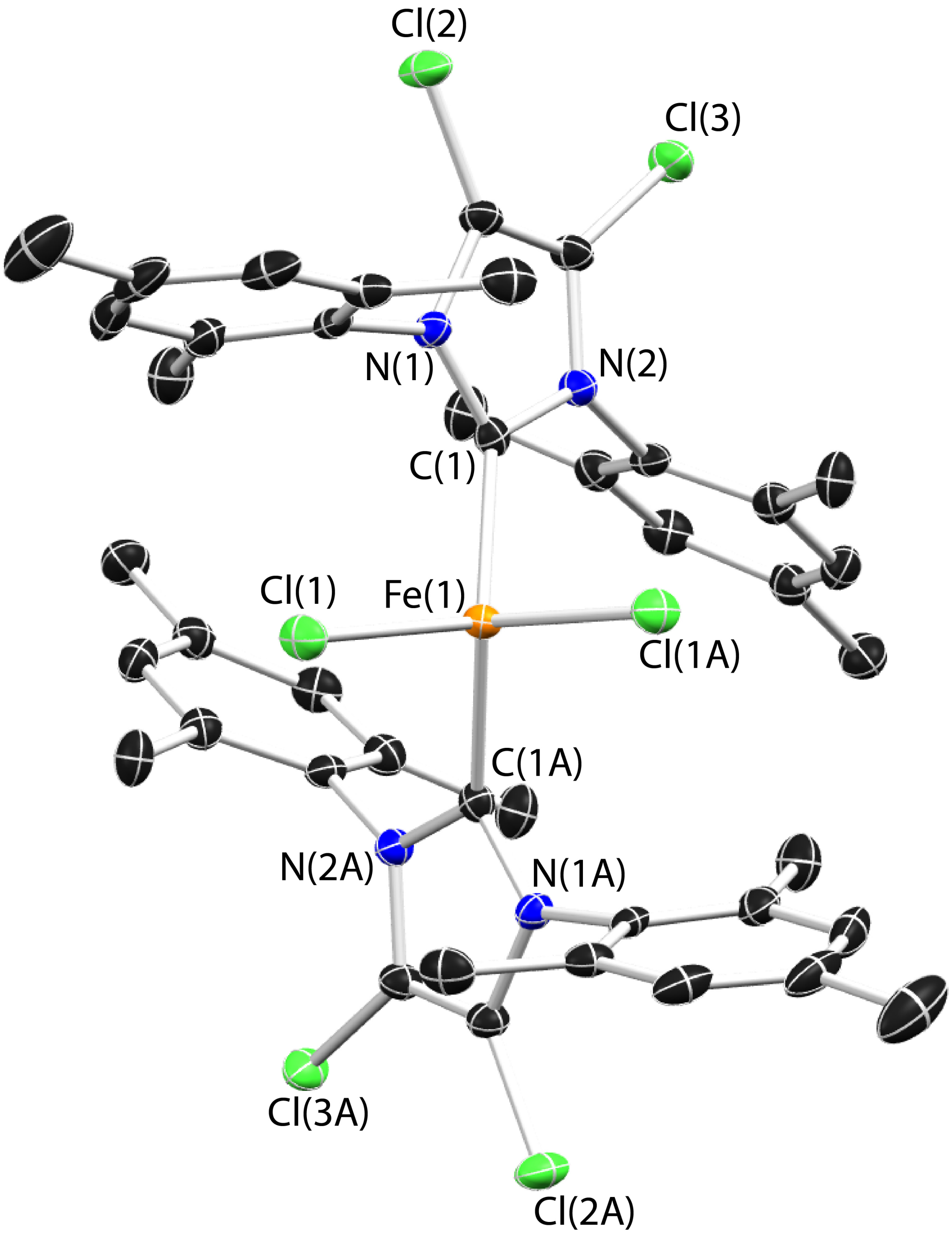

Figure S9. Thermal ellipsoid drawing (50%) of the solid-state structure of (^{Cl}IMes)$_2$FeCl$_2$. Hydrogen atoms omitted for clarity. Selected bond distances (Å) and angles (deg): Fe(1)-C(1) = 2.147(3); Fe(1)-Cl(1) = 2.2785(8); C(1)-Fe(1)-C(1A) = 124.20(15); Cl(1)-Fe(1)-Cl(1A) = 106.89(5); C(1)-Fe(1)-Cl(1) = 111.21(8).

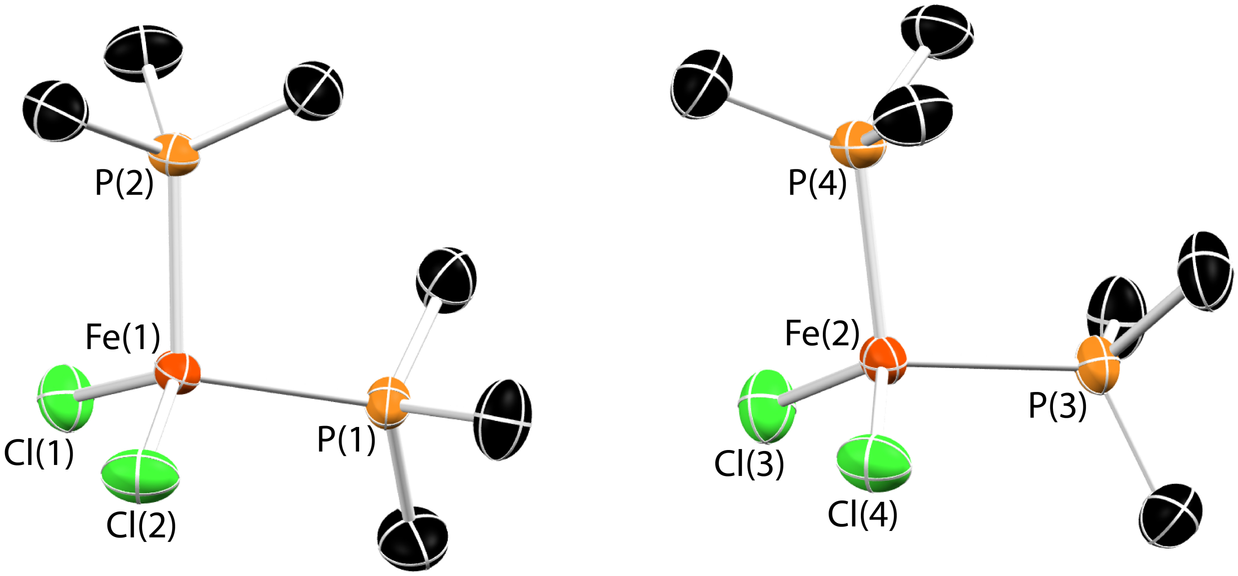

Figure S10. Thermal ellipsoid drawing (50%) of the asymmetric unit of $(PMe_3)_2FeCl_2$ displaying both crystallographically independent molecules. Hydrogen atoms omitted for clarity. Selected bond distances (Å) and angles (deg): Fe(1)-P(1) = 2.4267(5); Fe(1)-P(2) = 2.4302(5); Fe(1)-Cl(1) = 2.2355(5); Fe(1)-Cl(2) = 2.2392(5); Fe(2)-P(3) = 2.4341(5); Fe(2)-P(4) = 2.4306(5); Fe(2)-Cl(3) = 2.2399(5); Fe(2)-Cl(4) = 2.2348(5); P(1)-Fe(1)-P(2) = 102.802(13); Cl(1)-Fe(1)-Cl(2) = 122.879(18); P(1)-Fe(1)-Cl(1) = 103.419(17); P(3)-Fe(2)-P(4) = 102.095(14); Cl(3)-Fe(2)-Cl(4) = 123.30(2); P(3)-Fe(2)-Cl(3) = 106.605(18).

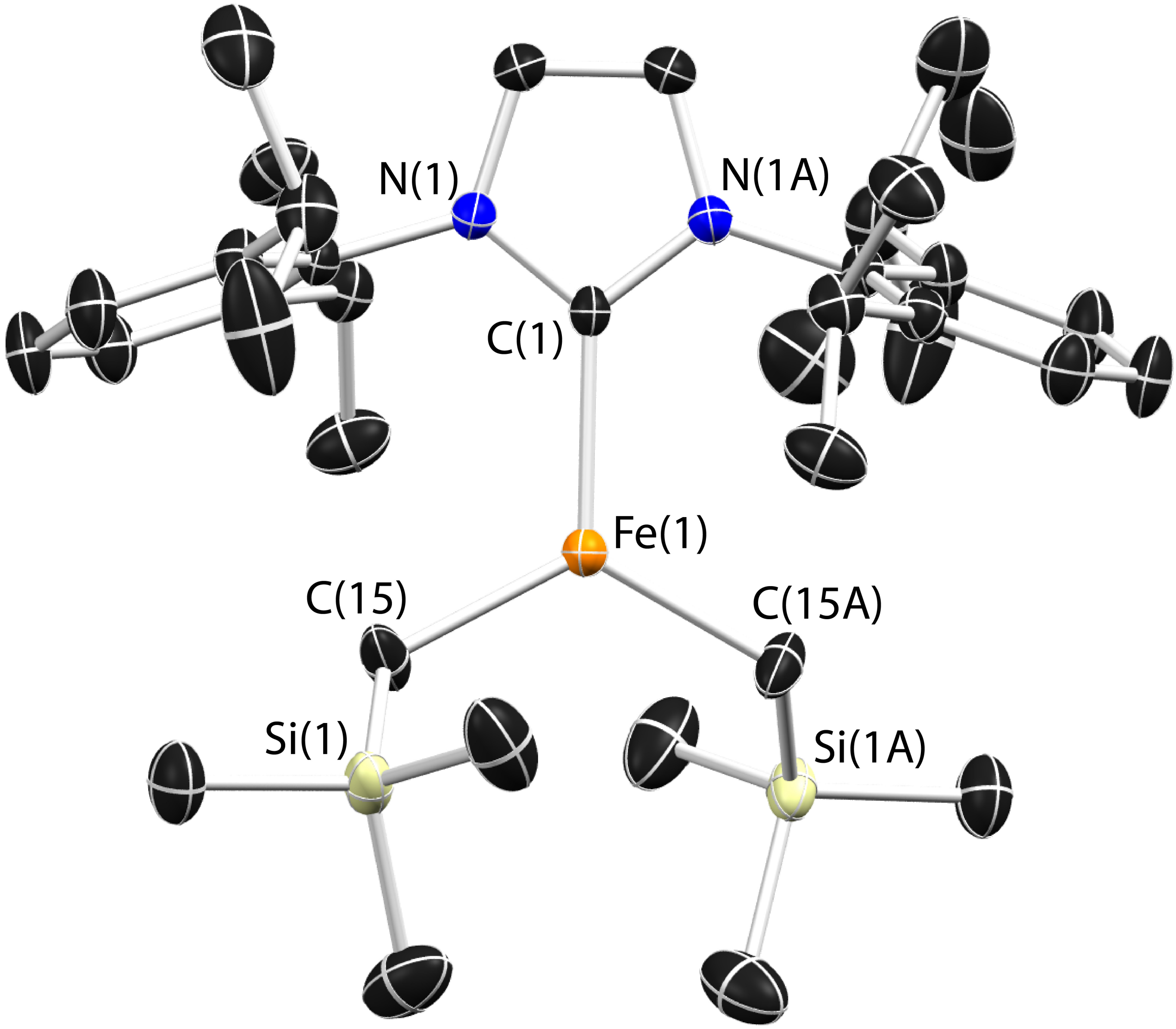

Figure S11. Thermal ellipsoid drawing (50%) of the solid-state structure of (IPr)Fe(CH_2TMS)$_2$. Hydrogen atoms and minor components of the disordered isopropyl group omitted for clarity. Selected bond distances (Å) and angles (deg): Fe(1)-C(1) = 2.164(3); Fe(1)-C(15) = 2.061(2); C(1)-Fe(1)-C(15) = 118.45(6); C(15)-Fe(1)-C(15A) = 123.09(13). ∑∡(Fe) = 359.99º.

Table S10. Crystallographic data and refinement parameters.‡

**Compound**	$(^{Cl}IMes)_2FeCl_2$	$(PMe_3)_2FeCl_2$	$(IPr)Fe(CH_2TMS)_2$
Empirical formula	$C_{42}H_{44}Cl_6FeN_4$	$C_6H_{18}Cl_2FeP_2$	$C_{35}H_{54}FeN_2Si_2$
Formula weight (g/mol)	873.36	278.89	614.83
Temperature (K)	98(2)	223(2)	98(2)
Crystal system, space group	Orthorhombic, *Fdd*2	Triclinic, $P\overline{1}$	Monoclinic, *C*2/*c*
Unit cell dimensions (Å, deg)	*a* = 19.230(2)<br>*b* = 43.035(5)<br>*c* = 10.2776(12)	*a* = 7.6475(8)<br>*b* = 12.4439(12)<br>*c* = 15.3186(15)<br>α = 102.743(2)<br>β = 95.406(2)<br>γ = 101.351(2)	*a* = 10.777(6)<br>*b* = 19.521(11)<br>*c* = 18.542(12)<br>β = 103.339(12)
Volume ($Å^3$)	8505.4(17)	1379.6(2)	3796(4)
Z	8	4	4
Calculated density (g/cm^3)	1.364	1.343	1.076
Abs. coefficient (mm^{-1})	0.766	1.666	0.483
F(000)	3616	576	1328
Crystal size (mm)	0.33 × 0.18 × 0.15	0.40 × 0.30 × 0.20	0.40 × 0.25 × 0.15
Θ range	2.66 to 26.50º	1.377 to 38.737º	2.09 to 27.50º
Limiting indices	$-24 \le h \le 18$,<br>$-18 \le k \le 54$,<br>$-12 \le l \le 10$	$-13 \le h \le 13$,<br>$-21 \le k \le 21$,<br>$0 \le l \le 26$	$-14 \le h \le 13$,<br>$-25 \le k \le 25$,<br>$-23 \le l \le 24$
Reflections collected / unique	5942 / 3408 [R_{int} = 0.0336]	84868 / 14854 [R_{int} = 0.0339]	14159 / 4360 [R_{int} = 0.0355]
Completeness to Θ	99.1%	94.2%	99.7%
Absorption correction	ABSCOR	multi-scan	ABSCOR
Min. and max transmission	0.681 and 1.000	0.5863 and 0.7476	0.827 and 1.000
Data / restraints / parameters	3408 / 1 / 240	14854 / 0 / 212	4360 / 0 / 203
Goodness-of-fit on F^2	1.018	1.020	1.017
Final R indices [I > 2σ(I)]	R_1 = 0.0349,<br>wR_2 = 0.0921	R_1 = 0.0338,<br>wR_2 = 0.0767	R_1 = 0.0485,<br>wR_2 = 0.1261
R indices (all data)	R_1 = 0.0355,<br>wR_2 = 0.0926	R_1 = 0.0563,<br>wR_2 = 0.0870	R_1 = 0.0462,<br>wR_2 = 0.1287
Δρ max and min ($e{\cdot}Å^{-3}$)	0.424 and −0.419	0.630 and −0.383	0.708 and −0.468

‡Refinement method was full-matrix least-squares on F^2; wavelength = 0.71073 Å. R_1 = aveF_o|–|F_c||/∑|F_o|; wR_2 = {∑=w(F_o^2–F_c^2)^2]/ FveF_o^2)^2]}^{½}.
